# A scoping review of the use of generative artificial intelligence tools in health profession education

**DOI:** 10.1186/s12909-025-08527-3

**Published:** 2026-01-23

**Authors:** Mounyah Basil, Waad Ahmed, Reem Hajeomar, Judith Strawbridge, Matthew Lynch, Banan Mukhalalati

**Affiliations:** 1 Department of Clinical Pharmacy and Practice, College of Pharmacy, QU Health, Qatar University, Doha, Qatar; 2 School of Pharmacy & Biomolecular Sciences- RCSI University of Medicine and Health Sciences, Dublin, Ireland

**Keywords:** Generative artificial intelligence, GenAI, ChatGPT, Large language model, Health professions education

## Abstract

**Background:**

Generative Artificial Intelligence (GenAI) is one of the leading innovations that is expected to reshape society for decades to come. Health professions education (HPE) programs are expected to prepare graduates with adequate knowledge and skills to provide high-quality patient-centered care. Although the use of GenAI in health professions is increasing, its optimal integration in HPE is still ambiguous. This scoping review aims to synthesize currently available literature regarding the use of GenAI in health professions education.

**Method:**

This scoping review is conducted following JBI methodology for scoping reviews framework 2020 and aligned with PRISMA-ScR. A systematic and comprehensive search was conducted in PubMed, ERIC, CINAHL, Embase, Scopus, Cochrane Library, and ProQuest Central with no language restrictions. The identified evidence was screened and extracted using Covidence software. Publications on the integration of GenAI in undergraduate or graduate health profession education were considered. Data was analyzed and presented using graphs and charts. Followed by a narrative thematic mapping of the included studies.

**Results:**

Out of 14,208 scanned records, 241 were considered eligible. The included studies discuss the application of GenAI in diverse education processes of different health professions, such as curriculum design, content creation, content delivery, personalized learning, assessment, evaluation, and feedback provision. Most studies focused on ChatGPT integration in medical and nursing education, with content creation emerging as the predominant area of integration, whereas curriculum design and GenAI literacy were underexplored. Perception studies reported a positive perspective regarding GenAI used in education among students and faculty.

**Conclusion:**

This review provides an overview of the current integration of GenAI in HPE in the literature, highlighting the associated opportunities, challenges, facilitators, and barriers. Future education efforts should focus on enhancing GenAI literacy, developing policy, and adopting a balanced approach. In addition to conducting comparative studies and long-term assessment of GenAI impact.

**Supplementary Information:**

The online version contains supplementary material available at 10.1186/s12909-025-08527-3.

## Introduction

In 1955, the term “Artificial Intelligence” (AI) was first introduced by John McCarthy in a Dartmouth workshop, where a proposal was submitted discussing the possibility of enabling machines to use language, solve problems, and improve themselves [1]. In the following years, AI has evolved significantly, impacting many industries such as the military, economy, entertainment, manufacturing, healthcare, medicine, and education [2, 3]. AI is a broad discipline, and multiple definitions are available to express its meaning. The European Commission (EC) High-Level Expert Group on AI defines artificial intelligence as “Systems that display intelligent behavior by analyzing their environment and taking actions, with some degree of autonomy, to achieve specific goals” [4]. Others define AI as a system that can analyze external information, learn from it, and apply this knowledge to achieve a specific aim in a flexible, adaptive manner [5]. Traditional AI consists of 6 main subfields, including Machine Learning (ML), Artificial Neural Network (ANN), Deep Learning (DL), Natural Language Processing (NLP), computer vision, and cognitive computing [6]. Generative Artificial Intelligence (GenAI) is an advanced technology that has evolved from traditional AI. GenAI relies on algorithms to analyze existing data (e.g., image and text) and then generate new, original, novel content [7]. It has a wide application due to the fact that it can generate not only text but also music, video, and images. Both traditional and generative AI are trained on large datasets. However, although GenAI relies on machine learning algorithms to analyze data, it does not follow predefined rules, unlike traditional AI [8]. Large Language Model (LLM) and Diffusion Model are both subsets of GenAI. Detailed definitions and categorization of both traditional and GenAI subtypes are provided in the Supplementary Appendix (Figure S1 and Table S1) [6, 7, 9–13]. 

Health professions education (HPE) encompasses advanced academic program in the field of medicine, dentistry, pharmacy, nursing, and other health-related professions [14]. AI is increasingly playing an essential role in enhancing education, as it shows the capability to overcome challenges and pioneer new teaching and learning methods. By decreasing routine workload, AI can enable educators to dedicate more time to continuous professional development [15, 16]. More importantly, AI is able to identify individual student differences in knowledge acquisition and thus generate a tailored, precise education based on individual needs, preferences, and experience, which is known as adaptive learning [15, 17]. 

Currently, the use of AI in health professions education in various processes, such as university admission, studying, teaching, and assessing performance, is relatively increasing [12]. However, such incorporation is still suboptimal, which comes with a dramatic increase in the demand to find the best way of integrating AI [12, 18]. This is especially true after the emergence of the Large Language Model (LLM), which forms GenAI platforms such as OpenAI’s ChatGPT on open access to the public. While OpenAI was established in 2015, it was not until 2018 that the early model showed a promising result. Masters has noted that GenAI has “caught HPE institutions off-guard” since the release of ChatGPT in late 2022 [7]. Since then, there has been a continuous necessity to develop policies on how to incorporate GenAI in education programs appropriately with careful consideration of its potential to give rise to ethical misconduct and plagiarism by both educators and students [7]. 

A scoping review published in January 2024 included a wide range of both original studies and perspective articles regarding the use of AI in undergraduate, graduate, and continuous professional development in medical education. The study does not address the application of AI in any health profession education program other than medicine. In addition, GenAI was not the main focus of this article [19]. To date, four scoping reviews have been conducted to specifically assess the use of GenAI in education, and one systematic review that examined HPE students’ use of GenAI in self-directed learning [20]. Two of the scoping reviews focused on a specific discipline such as medicine [21] or pharmacy [22]. Another review summarized the use of GenAI broadly in higher education for assessment only [23]. The fourth scoping review assessed the use of only ChatGPT, excluding other GenAI applications [24]. As the time frame for its search was up to November 2023, an updated study was necessitated due to the fast update rhythm of AI innovations and research [25], and the launch for public use in May 2024 of ChatGPT’s new version (ChatGPT4o). ChatGPT 4o has the advantages of image analysis, voice conversation, and more precise responses [26, 27]. 

Unlike systematic reviews, scoping reviews are considered flexible and inclusive in terms of study selection, which makes them appropriate for broad and complex questions [28]. Currently, there are no published or ongoing scoping or systematic reviews that comprehensively summarize GenAI technology use across a range of health professions education programs. Thus, this scoping review was conducted with the aim of synthesizing and providing an insight into the available literature regarding the use of GenAI in different health profession education programs. In addition to addressing a current gap in the literature, the primary objective was to explore how GenAI is used in various health profession educational programs globally. The secondary objectives focused on identifying the opportunities, challenges, facilitators, and barriers associated with GenAI integration.

## Method

The Joanna Briggs Institute (JBI) methodology for scoping review 2020 [29, 30], which is a modified version of Arksey and O’Malley framework [31] was followed to conduct the scoping review. JBI framework includes nine stages as follows [30].

- Stage 1: Defining and aligning study question and objectives.
- Stage 2: Specifying and aligning the inclusion criteria with the question and objective.
- Stage 3: Developing a plan for evidence searching, evidence selection, data extraction, and presentation.
- Stage 4: Exploring the evidence.
- Stage 5: Choosing relevant evidence.
- Stage 6: Extracting relevant data from the selected evidence.
- Stage 7: Analyzing the extracted data.
- Stage 8: Presenting the result.
- Stage 9: Summarizing the result, with a conclusion and future implications.

The Protocol was developed following the Best Practice Guidance and Reporting Items for the Development of Scoping Review Protocols [32]. Although the protocol stated that no restriction would be applied to the study design, commentary, editorial, reviews, and systematic reviews were excluded. Commentary and editorial were excluded because of the limited information and insight that they would add. Furthermore, reviews were excluded as GenAI is a rapidly evolving field that necessitates focusing on innovative studies. In addition, all the cited studies in those reviews were identified by the search strategy and were assessed against the eligibility criteria. The protocol was registered in the Open Science Framework (OSF) available at: (10.17605/OSF.IO/RHDJ5). The study also aligns with reporting standards of the Preferred Reporting Items for Systematic and Meta-analysis extension for Scoping Reviews (PRISMA-ScR) [33] presented in Supplementary Appendix 2.

### Stage 2: specifying the inclusion criteria

- Inclusion Criteria:
  - ◦ Studies that discussed the use of GenAI (LLM, diffusion model) in HPE.
  - ◦ Studies that discussed any of the following: opportunities, challenges, facilitators, or barriers of using GenAI in HPE.
  - ◦Studies conducted in any undergraduate or graduate up to licensure health profession education program (e.g., medicine, nursing, pharmacy, dentistry, veterinary, health science or allied healthcare).
  - ◦ Studies that discussed or described the use of GenAI in any of the education processes (curriculum design, teaching strategy, studying, assessment, or feedback provision).
  - ◦ Publication in any language
- Exclusion criteria:
  - ◦ Studies that discussed the use of non-generative AI forms (ML, DL, NLP, ANN).
  - ◦ Studies that discussed the use of AI in general without specifying GenAI.
  - ◦ Studies conducted in non-health-related professions.
  - ◦ Studies conducted in healthcare practice.
  - ◦ Studies conducted post-licensure in continuing professional development (CPD).
  - ◦ Studies that assessed the GenAI performance in solving exams with no further implementation or recommendation for use in HPE.
  - ◦ Studies that were published before 2018.
  - ◦ Editorial, commentary, and reviews

### Step 3: search strategy

The search strategy was conducted in PubMed, ERIC, CINAHL, Embase, Scopus, Cochrane Library, and ProQuest Central. Furthermore, a reference list of the included studies was screened for additional studies. ProQuest and ERIC include dissertations and theses, conference proceedings, and reports, which are considered grey literature. The search strategy evolved from an iterative pilot search conducted initially in PubMed and CINAHL. The comprehensive search strategy (keywords and index terms) was revised by a librarian and an expert in the field of AI and HPE (Supplementary Appendix 3). The final search was conducted on 31 st Jan 2025.

### Step 4 & 5: evidence screening & study selection

**Fig. 1:**
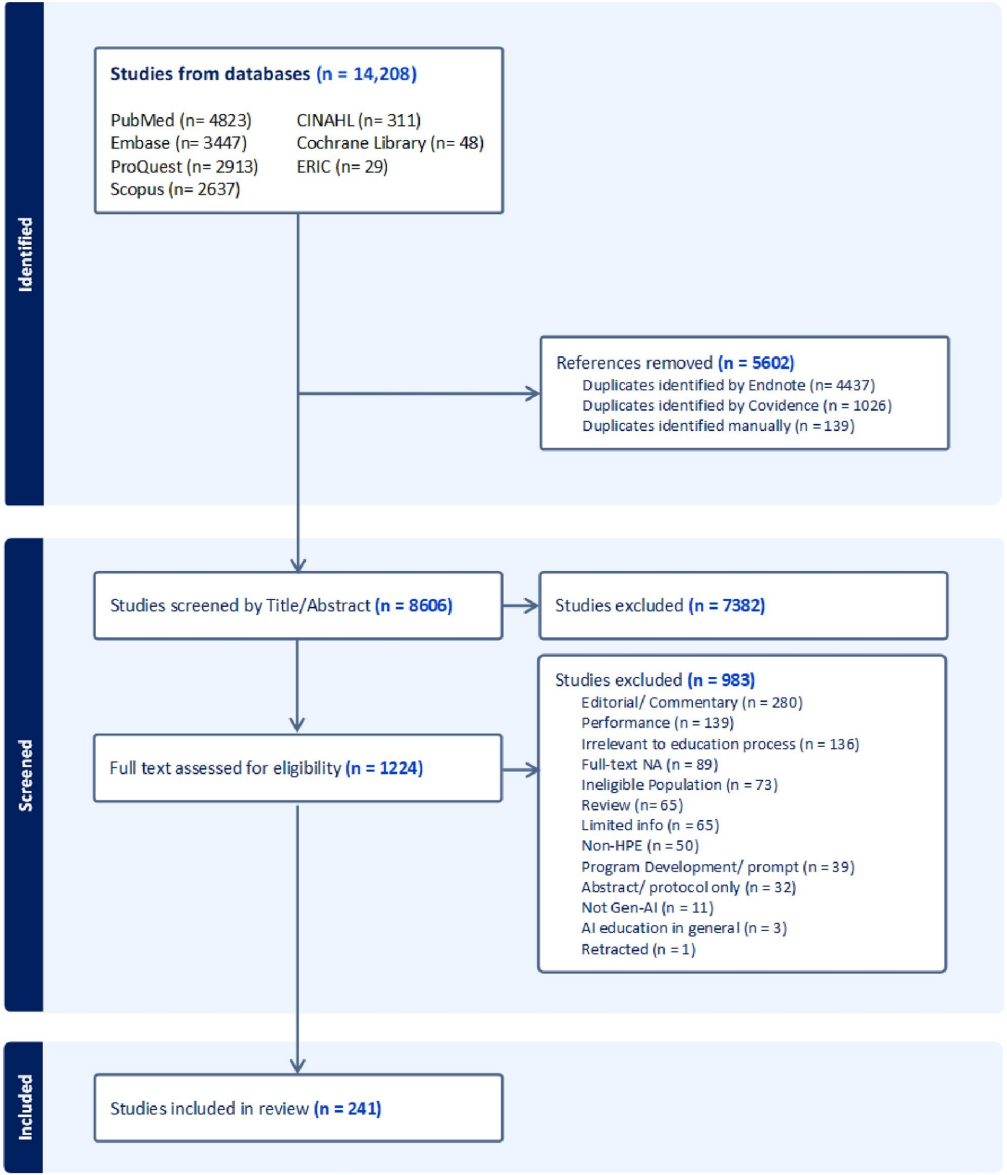
PRISMA diagram

All the identified studies from the databases were imported into EndNote 21 (Clarivate Analytics, PA, USA) accompanied by removing the duplication. Following that, screening and study selection was conducted using Covidence systematic review software (Veritas Health Innovation, Melbourne, Australia). At least two reviewers independently screened the titles, abstracts and then assessed the full texts for eligibility. Discrepancies between the reviewers were resolved through discussion or input from a third reviewer. Evidence screening, selection, and reason for exclusion were outlined in the PRISMA flow diagram (Fig. 1).

### Step 6: data extraction

Data extraction was conducted independently by at least two authors using a predefined and piloted data extraction form (Supplementary Appendix 4). The used data extraction tool was adapted with modifications from the published JBI data extraction tool and a published scoping review with a similar concept [29]. and a published scoping review with a similar concept [7, 29]. 

Data extracted from each study was as follows:

- Study identifier (study ID, title, author, year, journal).
- Study characteristics (type of publication, study design, country of origin, institution name (if available), aim).
- Population Characteristics (HPE discipline, specialty (if applicable), education level, sample size).
- GenAI (name of GenAI platform used, rationale for using GenAI, GenAI used by (administrator, instructor, student, researcher), GenAI applied for (e.g. curriculum design, content creation, assessment, etc.), implementation duration, how GenAI was implemented).
- Result (outcome of interest, tool used to assess outcome, summary of outcome, weakness/disadvantages of the used GenAI).
- GenAI opportunities, challenges, facilitators, and barriers.
- Implications for future educational practice, policy, or research.
- The study limitations as reported by the authors.

### Step 7 & 8: data analysis and presentation

All identified eligible studies were included in the data analysis and presentation. Data was summarized in graphs, charts, and tables to aid visualizing the distribution of resources by research method, country of origin, discipline, education step, etc. This was followed by a narrative summary categorized by educational process, opportunity, challenges, facilitators, barriers, and future implications.

## Results

The database search resulted in 14,208 publications. Following de-duplication, 8606 publications were screened for title and abstract, yielding 1224 publications for full text assessment. Of them, 983 articles were excluded resulting in a total of 241 publications for inclusion, with an average inter-rater reliability indicating substantial agreement (κ = 0.78). Screening process and reason for exclusion are illustrated in the PRISMA flow diagram (Fig. 1). Full data extraction using the developed tool can be found in Supplementary Appendix 4.

**Fig. 2:**
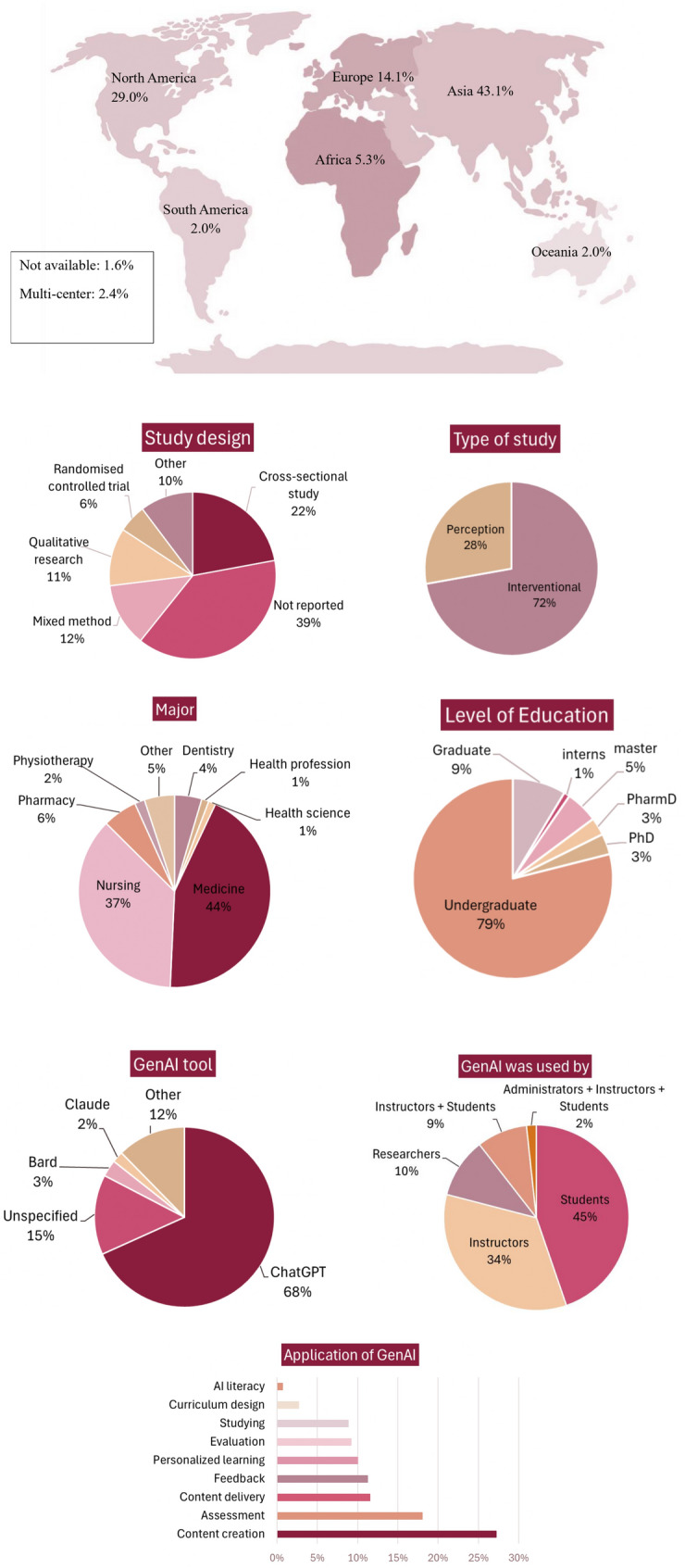
Infographic of the included studies

The infographic in Fig. 2 summarizes the included studies, including the regional distribution, study design, targeted health profession programs and their education level.

The included studies were distributed across various regions, with the majority conducted in Asia (43.1%), followed by North America (29.0%), Europe (14.1%), and Africa (5.3%). The study designs included cross-sectional studies (22%), mixed methods (12%), qualitative studies (11%), and RCTs (6%). Although 39% of articles did not report on their design, around half of them were classified based on the available information as follows: case reports, cross-sectional, non-randomized experimental studies, RCTs, pilot studies, and model development. The majority of studies were published in 2024 (72.1%), while the remaining were equally distributed between 2023 and 2025, with only one identified article published in 2022. The application of GenAI differed across the health professions programs, with medicine and nursing accounting for 43% and 36% respectively, with a greater focus on undergraduate studies (79%). Regarding the educational process, GenAI was commonly applied for content creation (26.2%), followed by assessment (18.0%), and to a lesser extent for curriculum design (2.7%), while only two studies looked at its application to improve GenAI literacy (0.6%). ChatGPT was the most frequently utilized tool, while others, such as Claude, Bard, Gemini, LLaMA, Copilot, and DALL-E were limitedly reported.

The studies included were categorized as either interventional or perception studies, as shown in Fig. 2. Interventional studies proposed specific applications of GenAI and evaluated their implementation within HPE in various educational processes categorized by themes. Perception studies investigated the viewpoint of students, faculty, or administrators regarding the integration of GenAI into education programs. Cited references in the following section are summarized in Supplementary Appendix 5, offering a cross-section of the full data extraction sheet which is provided in Supplementary Appendix 4.

### Interventional studies

#### Curriculum design

Eight studies have discussed the use of GenAI, particularly ChatGPT, in various tasks associated with curriculum design. In two studies, ChatGPT was embedded into a structured instructional design models- DRIIPT (Define, Research, Interpret, Ideate, Prototype, Test) [34] and CIDI (Clarify, Ideate, Develop, Implement) [35]. In planning a “Healthcare Design Thinking” course, ChatGPT was integrated into each DRIIPT framework aspect that involved analyzing existing curricula and identifying gaps. In addition to creating learning activities aligned with the intended learning outcomes, it supported formulating and visualizing the course components (e.g., timeline, syllabus, and storyboard), piloting the developed course, and refining it based on simulated stakeholders’ feedback. Overall, medical students were satisfied with the delivered course in terms of its relevance and consistency [34]. Similarly, ChatGPT was integrated into the CIDI model as an assignment for nursing students to design a health education lesson plan, which enhanced the students’ critical thinking, problem-solving, and learning enjoyment [35]. Many studies utilized GenAI (e.g. ChatGPT, Claude, Gemini) to generate and revise learning objectives following Bloom’s or Flink’s Taxonomy, and to plan learning activities and assessment questions guided by the learning objectives [34, 36–38]. The performance of GenAI in such aspects was assessed to a limited extent by only one study, finding that GenAI produces more relevant learning objectives when prompted with a small, focused content, and that only 38% of GenAI output can be used without modification [36]. It also played a role in curriculum adaptation by assisting administrators and instructors in adjusting lecture duration and teaching plans based on the topic difficulty and student performance [39–41].

#### Content creation

Utilizing GenAI for content creation was reported in 80 studies; however, the integration varied significantly based on the aim and the desired outcome. In some studies, cases were generated by researchers or instructors for assessment or education purposes, whereas in others, students used GenAI to develop case reports and care plans as part of their learning process [42, 43]. The GenAI-generated clinical cases complied with ethical standards and effectively identified and explained the ethical concerns in the generated cases [44]. One study showed that students who employed ChatGPT and Copilot to write and modify their case report assignment achieved significantly higher assessment scores, GenAI literacy, self-efficacy (learning new AI skills and solving AI-related problems), and self-competency (emotional regulation) when compared with those that did not. Although Copilot’s output was satisfactory, ChatGPT provided a more comprehensive explanation [42]. Other studies investigated the ability of GenAI to generate clinical case material in languages other than English [42, 45, 46]. For instance, of the GPT-4-generated cases, 58% were medically accurate and 70% were linguistically correct (Japanese) to be used immediately with only minor modification [45]. 

Although the majority of studies highlighted GenAI benefits, two studies reported conflicting outcomes. In a pediatric course, nursing students were asked to use either ChatGPT or a textbook to solve a complex social case such as one involving child-welfare. The textbook group significantly outperformed the ChatGPT group in terms of critical thinking, clinical reasoning, utilizing evidence-based resources, the application of ethical principles, and the critical evaluation of ethical dilemmas and challenges. Although the ChatGPT group was allowed to use other resources, only 21% cited textbooks, and 9% referred to lecture notes or other search engines [47]. 

Aside from clinical case applications, content creation may include generating storytelling, images, comics, animated videos, and music. The University of Idaho in the United States developed a cinematic clinical narrative experience to enhance medical students’ engagement in the infection and immunity course. DALLE, a GenAI tool that converts text into images, was used in multiple studies to create visual educational content. Some images were highly relevant to the requested educational theme (e.g. pathophysiology, patient education, clinical trial recruitment, bullying), representing sensitive topics adequately, improving student understanding to complex concepts, and decreasing the time and expertise barrier associated with visual content design. Despite these benefits, some experts classified the majority of congenital heart disease images, generated using DALLE, as fabricated, anatomically incorrect, wrongly labeled, not beneficial to medical education, and unattractive, leaving only 0.4% to be usable with no modification [48]. 

#### Content delivery

GenAI tools were used for content delivery in 34 studies. The majority utilized GenAI to develop a simulated patient that interacts and communicates with students in role-play training. Some studies provide a more interactive experience by incorporating 3D avatars [49–51]. In one study, a virtual patient avatar named Sarah was generated with a complex medical case using the Convai platform for anesthesia training. Experts assessed the tool favorably, with a high rating for ease of use and intuitive design (score: 9/10), as well as for its accuracy in simulating patient behavior (8/10). Most experts (87%) were comfortable using this tool and perceived it as a feasible substitution for a standardized patient in an assessment setting. Nevertheless, certain limitations were noticed by the experts, including answer repetition, lengthy replies, and using very formal and polite language, which may not mimic real-world situations [49]. To address these issues, a study integrated multiple personalities (talkative, rude, polite, and ill-tempered) and supported the GenAI with a profanity filter to ensure language appropriateness [52]. GenAI robots were also utilized in two studies, and they outperformed the computer-based simulator by providing more authentic and realistic training. Interaction with such technology created a safe environment for students to train in medical history taking and critical reasoning skill development with no possible harm to actual patients. However, students were unable to perform lab tests or physical examination, and technical modifications were required as it misinterpreted students’ pauses, generating responses without being asked [53, 54]. A Metaverse Art Gallery of Image Chronicles (MAGIC) was created by using ChatGPT-3.5, NightCafe, Canva and HeyGen to deepen pharmacy students’ understanding of patient perspectives regarding their medication. This was done by personifying patient medication as villain or hero characters based on their experience. The portraits were then animated into videos and collected in the metaverse, where students can interact and engage within the generated story in different realms. Students reported improvement in their understanding of medication adherence, art therapy, and the patient’s relationship with medication. In addition, they expressed satisfaction and perceived the experience as enjoyable, useful, and suitable for their learning style [55]. 

#### Personalized learning

Employing GenAI to provide a tailored learning experience was discussed in 30 studies. In one study, medical students in a pharmacology course used ChatGPT as a pre- and post-class assistance to clarify complex concepts (e.g. pathogenesis theory) and compare closely related topics (median effective dose (ED50) Vs median lethal dose (LD50). Moreover, it generated and analyzed patient scenarios, treatment plans, and monitoring strategies with cross-chapter integration. ChatGPT provides a more student-friendly, detailed, and structured response when compared to a search engine and medical textbook, which enhances knowledge and critical reasoning skills [56, 57]. A study randomized medical students to either a traditional learning group or a ChatGPT-based blended teaching. Embedding GenAI in the learning process significantly improved student satisfaction, clinical skills, and performance in the theoretical exam compared to traditional learning [58]. On the other hand, some interventions focused on improving certain skills, such as writing [59] or speaking [60]. Students used ChatGPT to practice their English-speaking skills individually for over 7 weeks, as it generated different activities such as role-playing, storytelling, and simulated interviews. The intervention significantly improved the speaking test score, reduced student anxiety, and alleviated feelings of low confidence, hesitancy, embarrassment, and a fear of participating in oral activities [60]. In addition, students utilized ChatGPT in an academic writing course to proof-read their weekly assignments and edit them, if necessary, with a justification for each modification. Ultimately, students using ChatGPT showed a significant improvement in their academic writing skills when compared to those who relied on traditional methods to achieve this. ChatGPT enhanced all aspects of their writing, including content, vocabulary, conventions, and organization, although no difference was reported in the language used [59]. 

#### Assessment

GenAI has been used to ease the creation of assessment elements with consistent standards of quality in 53 studies. Compared to the time required to generate MCQs by educators (30–60 min), ChatGPT was able to produce MCQs in a shorter duration (5–15 min), but all required editing before use. Furthermore, the distractors used by ChatGPT were not always relevant to the content taught, thus requiring editing. Despite the need for edits, student performance in ChatGPT MCQs and non-AI MCQs was similar, with no differences in the discrimination value. Several advantages were reported, such as enhancing efficiency, reducing cognitive burden on instructors, and decreasing the time required to generate and reference content. Thus, teachers were able to focus on the higher-level editing of items created [61]. In addition, GenAI was rated by examiners as equally realistic and appropriately difficult in terms of generating clinical scenarios used for formative assessment. It significantly reduced drafting time (576 person-min vs. 1190 person-min) and the financial cost when compared to the traditional method (Stg£652 vs. Stg£1530) [62]. However, GenAI outputs’ quality differs among various medical specialties. For instance, ChatGPT was used to generate MCQs with neurology questions had fewer item-writing flaws (5%) when compared to endocrinology questions (16%)[63]. Another study revealed that ChatGPT, and Claude generated Objective Structured Practical Examination (OSPEs) assessment items were technically accurate, free of construction defects, comprehensive, and suitable for the learner level. Both GenAI tools provided model answers with explanatory notes that can facilitate formative assessment for both learners and instructors [64]. 

#### Evaluation

Of the identified studies, 27 involved the use of GenAI for evaluation, primarily focused on comparing GenAI evaluation to human evaluation. Several applications of GenAI in evaluation were addressed, including grading clinical notes/reports, assignments, essays, reflections, and student answers to given questions. Students’ clinical notes from a role-play case scenario were assessed by a standardized patient and ChatGPT based on predefined rubric. ChatGPT-3.5 provided an error rate of 1.0% which was significantly lower than standardized patients (7.2%), with an 86% reduction in errors, suggesting its capability to accurately grade [65]. In contrast, a study found that the agreement between the GenAI methods and standardized patient evaluators varied significantly, from 26% to 83% [66]. Another study showed GPT-4 grading of students’ clinical reports during OSCE to be stricter when compared to a human grader, with an average lower score of 3.51, mainly due to it adhering more closely to checklist criteria [67]. These various results highlight areas for future improvement in using GenAI in assessments.

Another application is in automated essay scoring (AES). A study found that ChatGPT-4 evaluated dental students’ assignments and provided detailed feedback within a shorter duration than human tutors (2 days vs. 5 days), thus reducing the workload on educational assessors [68]. Some graders utilized GenAI-assisted grading to inform their assessment and validate their judgment accuracy [69]. Another study showed that GPT-4 gave lower scores when compared to human evaluators’ grades, while Gemini 1.0 Pro was shown to be more comparable. It was also noted that LLMs often demonstrated a reluctance to score extreme grades (either 0 or full marks) [70]. 

#### Feedback

Providing feedback is one of GenAI applications that was highlighted in 34 studies. For instance, researchers developed iAtexF GenAI, to provide real-time feedback to physiotherapy students while they conducted a physical examination. iAtexF generated clear, structured, and accurate feedback, surpassing ChatGPT or experts in providing structured, organized feedback with relevant suggestions for improvement in a positive tone [71]. In another study, GPT-4 was used to evaluate students’ answers and provide feedback on antibiotic stewardship education, where the feedback significantly improved student knowledge. In addition, experts classified 92% of the generated feedback as “Helpful” with no identified hallucination (false and fabricated information) [72]. GPT-4 was also used to deliver live feedback for dental and nursing students interacting with virtual patients, helping them develop empathy and communication skills. GPT-4 generated feedback significantly improved students’ empathy when compared to the control group, together with a positive perception of both the feedback’s accuracy and helpfulness [73]. Another study reported that GPT-4 generated realistic and flexible feedback, which improved students’ confidence and interview skills [74]. Furthermore, the majority of students who received ChatGPT feedback on their SMART goals reported ongoing use of the tool at 2 weeks post-intervention [47]

### Perception studies

Many studies investigated students’ perspectives and current utilization of GenAI in education. The frequency of GenAI use varied across studies; in some, around 20% of students reported using the tool daily to weekly for academic purposes [75–77], whereas in two studies, usage exceeded 40%.^(78, 79)^ Primary uses were very broad and depended on the student discipline and the university regulations. In general, Medical and pharmacy students reported more frequent use of GenAI tools in their assignments, writing personal statements or proposals, revising and editing text, and conducting research projects [79–81]. However, they were unlikely to use it in writing a clinical note or as an information source, especially in a clinical setting [79, 82]. This contrasts with PharmD students and Midwifery students who intensively utilized GenAI tools for obtaining disease and drug information [82, 83]. In addition, they employed ChatGPT to develop care plans, both pharmacological and non-pharmacological, and to answer drug information questions [81, 82, 84]. Other health professions, including nuclear medicine, dentistry, and nursing students utilized the tool for active learning strategies such as flipped classroom, case-, team-, and problem-based learning [85–87]. Educators most commonly leveraged GenAI for gaining knowledge, conducting research, and assisting in clinical decision-making [88–91]. In addition, the tool was used for generating teaching resources, developing and updating lecture notes and slides, formulating lesson plans, drafting assessment questions, assignments, and generating virtual cases [89–91]. Stakeholders utilized it for administrative tasks such as drafting and proofreading policies and procedures [91]. 

Students reported perceiving advantages of GenAI including finding drug-related problems, ADR, drug-drug interactions [82], enhancing the delivered care, diagnosis, and treatment selection [92]. GenAI can also improve patient safety by performing accurate medical calculations and providing educational material tailored to their level of understanding [93]. In addition to saving time, providing fast access to a comprehensive information pool facilitated the learning process and improved academic performance [83, 85, 90, 93–96]. Breaking down language barriers and improving the learning of foreign languages were also reported [83, 90, 93, 95]. In addition, GenAI can improve the quality of writing by proofreading, correcting grammar, and providing and appraising resources [93, 95]. In clinical rotations, GenAI enhanced students’ critical thinking, communication, engagement with the health care team, ability to provide comprehensive care, and perform stewardship activities [97]. Furthermore, GenAI improved ethical literacy, ensures equitable distribution of learning resources, and supports topics with limited resources [86]. Cross-checking AI-generated content was reported in one study, where only 18% of students never verified LLM output [98]. 

Prior knowledge and exposure in using GenAI had a significant impact on student behavior in terms of tool utilization, cross-checking, and current and future trust in the generated information and clinical decision-making ability [98]. In one study, only 5% of students had been educated about GenAI, while GenAI ethics education was received by only 4.3% of students [92]. Several factors can influence GenAI intention to use, including personal innovativeness, using behavior, facilitating conditions, social factors, perceived usefulness, ease of use, and expected risk [78, 94]. Age group also impacted GenAI familiarity, comfort level, understanding, and using patterns, with younger students being better in all aspects [93, 99]. On the other hand, education stakeholders were reluctant to integrate GenAI into educational practices, citing concerns about patient perspective, effect on society, and academic integrity,. In addition they expressed apprehension regarding output accuracy, ethical issues, lack of support from institutions and leadership, and uncertainty regarding its applicability and the potential benefit [91]. This contrasted with students who were enthusiastic due to the potential of decreased financial burden, time savings, and increased efficiency [80]. Some studies reported that male students had a higher usage rate, a more optimistic attitude, greater trust, and better understanding of the potential benefits and limitations of GenAI utilization [99, 100], while usefulness was perceived more strongly by females [101]. In addition, non-native speakers reported more frequent and confident use of GenAI compared to those students who were natives [100]. 

Integrating GenAI into the educational process was assessed by some studies, with the majority of students supporting GenAI incorporation in the study curriculum [80, 98, 102]. Some students reported receiving faculty encouragement to utilize GenAI for educational purposes [80], while the majority stated ambiguity in college professors’ and clinical preceptors’ opinions regarding the use of GenAI [75, 84]. One cross-sectional study explored physical therapy program directors, deans, department chairs, and faculty’s perspectives regarding GenAI integration. While 54% reported nervousness about its use in higher education, 42% were optimistic, and 75% believed that it will reshape higher education in the upcoming 3–5 years. Only a third thought that GenAI benefits outweighed the risks and that it might ease their job [91]. 

### Barriers, Facilitators, Opportunities, and Challenges

**Fig. 3:**
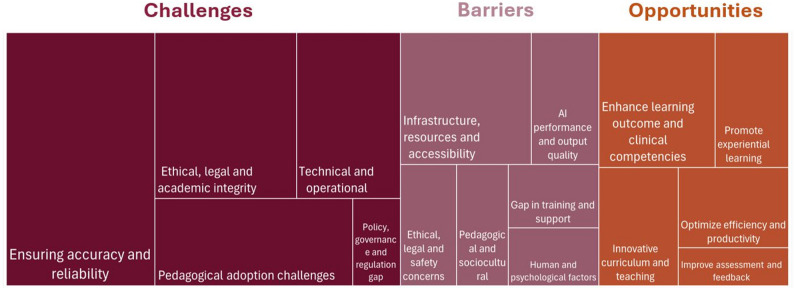
Treemap of Challenges, Barriers and Opportunities

Studies included in this scoping review revealed that the implementation of GenAI in HPE is influenced by a number of complex factors. The identified facilitators, barriers, opportunities, and challenges provided insight into the multifaceted nature of implementing GenAI in HPE. Figure 3 presents a hierarchical visualization of the more prevalent themes in challenges, barriers and opportunities reported in the included studies.

Four studies reported facilitating factors that aided GenAI implementation in HPE. In one study the institution provided a reimbursement scheme covering the subscription fees on ChatGPT-4 Turbo for one month for any student; thereby removing any financial barrier [103]. A customized GPT with Johari Window and Crucial Conversations Framework for medical students was also made free of charge to facilitate its use [104]. In addition, nursing students mentioned that the GenAI had user-friendly interfaces, free access, and ease of use which supported its integration into the academic setting [105]. In another study, a customized mobile app called AppER that used GPT-4-turbo for generating feedback for medical students was developed. The Firebase was utilized to manage the database, where all items were stored, and facilitate multiple cloud functions, such as sending notifications and processing data [106]. 

On the other hand, multiple barriers to GenAI implementation were identified, with infrastructure, resources, and accessibility emerging as the most frequently reported factors. Infrastructure issues ranged from inadequate internet connectivity and access to technology, power interruptions, and country restrictions requiring the use of VPN [77, 86, 107]. Students from different health disciplines reported socioeconomic barriers that impeded GenAI accessibility, such as device availability, internet connectivity, and the need for assistant support for people with disabilities [108]. The other study found that students in South Africa can face scheduled electricity outage (load shedding), high data costs, and poor infrastructure, which impacted their utilization of GenAI [107]. Cost was repeatedly stated as some advanced models required a paid subscription (e.g. ChatGPT Plus and DALLE-3), which could be a burden on low-income countries [109, 110]. Additionally, language and cultural differences also limited the usability of GenAI, especially in multilingual settings [107, 108, 111–113]. GenAI performance and output quality were also identified as major barriers; for example, faculty who used ChatGPT to generate clinical cases noticed inaccuracy and inconsistency in the generated content such as mismatching family histories, medications, or physical exam findings [114]. Similarly, students reported that GenAI sometimes generated inaccurate or incorrect references and the possibility of logical errors [115]. Other reported poor performance of GenAI in generating complex clinical scenarios, especially tasks that required advanced knowledge and experience [116]. Another technical issue was the functional gap in image and data interpretation. Two studies discovered that ChatGPT cannot correlate the generated text to clinical photos and radiology images, and lack mathematical calculation ability [115, 116]. Other barriers were associated with certain GenAI applications, specifically those designed as virtual patient (VP) or metaverse realms, where the lack of physical examination and laboratory testing hindered the clinical reasoning training [53]. Ethical issues arising from the use of GenAI were also perceived as a barrier. Students believed that GenAI posed bias risks, plagiarism, and promoted cheating. Further, it might give rise to confidentiality concerns because of data privacy and security concerns, arising legal implications [101, 117]. Human and psychological hindered GenAI integration, including fear of potential misuse and overreliance, as well as a preference for traditional learning approaches [88, 107, 118–121]. Training gaps were identified as a barrier to GenAI adoption, where limited preparedness affects educators’ knowledge and skills regarding GenAI [107, 119]. 

Despite the barriers, the literature reveals significant opportunities that GenAI presents for transforming healthcare professional education. GenAI creates clinical vignettes, opening new education opportunities that improve students’ academic performance and cultivate their clinical skills and ethical literacy [45, 58]. In addition, it can build and generate clinical scenarios that simulate real-life encounters, providing the opportunity to practice in a safe and controlled environment, which increases students’ confidence and reduces anxiety [122, 123]. It can serve as a fountainhead of literary material and comprehensive source of knowledge, transforming didactic lecture into an interactive workshop, allowing more space for brainstorming and discussion [124, 125]. Comparing the GenAI response to real-world case reports can enrich discussion and foster analytical and critical thinking skills. For instance, medical students can benefit from evaluating ChatGPT output against the published literature in understanding how variation in patient presentation can influence the selection of acupuncture points [126]. 

HPE educators can benefit from GenAI to create various educational materials with less effort, such as developing lesson plans and learning modules, and drafting examination questions, assignments, and case studies. Thus, it supports adaptive learning by creating personalized study plans and learning materials, and using digital patients who are more diverse than real patients [90, 91]. GenAI generated and updated lectures, assisted in administrative writing tasks such as drafting emails, letters of recommendation, and other documents for the institution [91]. In addition, GenAI promoted remote education, which could enhance the interactivity and personalization of online learning that is especially beneficial in resource-limited settings [127]. Furthermore, it could facilitate research and publication, providing an opportunity to publish in a shorter time with better quality [57, 91]. Clinically, it could be useful in clinical decision-making or retrieving nursing and medical information, helping practitioners become more proficient [128]. 

However, alongside these promising opportunities, the implementation of GenAI also presents ongoing challenges that require careful consideration and management. The integration of GenAI into HPE is associated with accuracy and reliability concerns, including hallucination, outdated medical guidelines, and clinically inappropriate recommendations such as incorrect drug dosages or non-existent diseases [45, 48, 79, 93, 95, 107, 129–139]. Another aspect are the ethical risks, such as academic dishonesty, including plagiarism which impacts equity and creates unfair advantage to some students [93, 95]. Some academic institutions limited the access of GenAI to their resources for security concerns thus impacting its utility [61]. In education, overdependence on GenAI threatens the critical thinking process, leading to copy-and-paste behaviors that potentially bypass deep learning [93]. Technical challenges, such as inconsistent responses, word limit, and difficulties in processing long prompts, hinder its utilization [46, 69, 131] It also fails to critically appraise research articles with an inability to differentiate primary/secondary literature, which limits its use in evidence-based practice [140]. GenAI also lacks humanistic factors such as personal interaction and feedback; thus it cannot teach caring or compassion [72, 112]. Furthermore, GenAI demonstrated limited awareness of social determinants of health, and primarily relies on western values which may result in a content that does not account for diverse social and cultural context [95, 138]. 

### Implications for future educational practice, policy, or research

The included studies have suggested various recommendations to shape the future of education, research and policy development. These are presented in Table 1.

**Table 1:** Future educational practice, policy, and research

Educational Practice
1. Encouraging balance utilization to avoid overdependence[96, 98].
2. Directing students to critique AI-generated output with cross-referencing in order to improve students’ critical thinking and judgmental skills[42, 47, 56, 57, 141, 142].
3. Providing training programs (e.g., orientation, seminars, workshops) to enhance the competency of both faculty and students in effective and responsible use of GenAI in education[64, 75, 79, 91].
4. Developing a structured framework that facilitates and optimizes the prompt engineering process with continuous support from IT department[94, 114, 130, 141, 143].
5. Integrating GenAI into the education process, including designing curriculum, simulating patient interaction, and providing real-time feedback and personalized learning[42, 47, 73, 79, 83, 88, 92, 98, 139, 144, 145].
6. Developing grading rubrics to ensure consistent responses[146].
7. Integrating GenAI literacy and GenAI ethical education into the curriculum with hands-on experience and support from their libraries[88, 92, 119, 147].
8. Collaborating with academic communities on an ongoing basis to share the latest findings, best practices, and resources[88, 91, 148].

Policy
1. Developing policies and guidelines to regulate the use of GenAI in education while maintaining academic integrity[42, 82, 142, 145].
2. Collaborating with ethical experts and national institutions to develop GenAI ethical use standards and best practices[77, 91].
3. Piloting the ethical guideline developed in advance of publication and auditing it thereafter[40, 47, 91].
4. Evaluating and updating the GenAI policies periodically due to the fast evolution of GenAI technology[44, 112, 149, 150].
5. Addressing the infrastructure gap to ensure equitable access[40].
6. Establishing proactive strategies before integrating GenAI in HPE, including bias mitigation and risk management policies[93, 115, 151, 152].

Research
1. Conducting longitudinal trials to assess learning outcomes with larger sample sizes and more diverse populations[79, 83, 91, 94, 98, 140, 153–156].
2. Assessing the long-term challenges and facilitators associated with GenAI integration into the education process[145, 157]
3. Evaluating the accuracy and educational impact of the AI-generated content (MCQ, multimedia) and virtual tutor[61, 73, 158].
4. Refining the utilized prompt and establishing prompt modification strategies to ensure output consistency[64, 159, 160].
5. Comparing different GenAI models/applications with ongoing assessment of emerging tools[88, 156, 159, 161].
6. Investigating the effects of combining different GenAI tools[42].
7. Assessing the financial impact of GenAI integration by conducting a cost-benefit analysis[140].
8. Exploring students’ and educators’ perspectives of GenAI at the national level, with a focus on evolving stakeholder roles in this era[40, 79, 88, 93, 103, 162].
9. Strengthening the evidence base through comparative trials and mixed-method studies[42, 91, 94, 163–165].

## Discussion

This is the first comprehensive scoping review to synthesize all the available evidence regarding the integration of GenAI in education programs across a broad range of health professions to date. The extent of GenAI integration differs across disciplines, with medicine being the most frequently represented profession in the literature. ChatGPT emerged as the most widely utilized GenAI tool, followed by Google Bard. The application of GenAI in education encompasses a range of areas, including curriculum design, content creation, content delivery, personalized learning, assessment, evaluation, and feedback provision. Despite the anticipated benefit of GenAI in curriculum design, the current integration in HPE remains limited compared to other educational processes, accounting for only 2.2% of the identified studies. On the other hand, literature explored GenAI applications for curriculum design in other disciplines, particularly education, where English pre-service teachers (PST) used ChatGPT and Claude.ai to support lesson planning for middle and high school students [166, 167]. In these studies, PST reported that GenAI facilitated the generation of clear lesson plans and learning objectives and provided creative ideas and engaging activities with accurate time allocation. Moreover, GenAI supported the application of pedagogical principles and minimized the mental load and effort associated with repetitive tasks. However, the GenAI outputs have been criticized for their limited accuracy and reliability, as well as for the absence of student-centered activities that foster critical thinking. In addition, educators might become overly dependent on GenAI for producing educational material, necessitating a balanced approach that leverages its advantages while maintaining human oversight [166, 167], which is consistent with this review’s findings Although no barriers were associated with GenAI integration specifically in curriculum design in HPE literature, studies from other disciplines identified challenges such as word count limitation, restricted access to advanced features, and the need for iterative prompting and rephrasing the output [166, 167], which could explain the scarcity of evidence. Training educators on the effective and responsible integration of GenAI was consistently emphasized across all disciplines [64, 91, 96, 166–168]. Several sources of evidence across different educational levels indicated that improving GenAI literacy and the familiarity of educators with GenAI correlates with higher adoption in the educational context [169, 170]. 

Unlike curriculum design, the integration of GenAI into content creation has been widely reported in the cited evidence. The literature reviewed suggested that GenAI is capable of identifying student needs and preferences, thereby creating educational content that facilitates adaptive and personalized learning [171]. The extensive integration of GenAI in HPE programs observed in the retrieved trials was consistent with educators’ self-reported usage in real-world settings. Despite the extensive application of GenAI for content creation in the cited articles, the diversity of GenAI tools utilized was limited, mostly ChatGPT, except in studies that generate multimedia content. A number of specialized GenAI tools were utilized to generate videos, images, comics, and virtual realms for educational purposes, such as Midjourney, Night Cafe, HeyGen, Canva, Leonardo.ai, Eleven Labs, and Suno Chrip Bot [55, 153, 172, 173]. On the other hand, literature is available to guide the educator in the selection of appropriate GenAI tools for targeted educational purposes. Some articles presented a compilation of GenAI tools, for example, MagicSchool, Curipod, Eduaide.ai, Learnt, a.i., and teachology.ai are recommended for planning lessons, generating content, and designing activities, including Project Based Learning (PBL) and Inquiry-Based Learning (IBL). Other GenAI such as SLIDESGO, SlidesAI, Presentations AI, Desktopus, MotionAI, HeyGen and Runway facilitate video and slides creation [174]. While other literature comparing GenAI tools performance for content creation in education (not HPE specific) revealed that Grammarly Business and Scalenut were superior to TextCortex AI and Ryter [175]. 

Analyzing the included studies revealed that GenAI was also utilized in content delivery to simulate patient encounters with healthcare providers. The observed advantages of such integration closely align with those reported in the literature [176, 177], while also offering opportunities to train students in the management of rare diseases that can be challenging to secure in clinical placements [178]. Furthermore, other literature suggested that GenAI is effective in supporting Social Emotional Learning (SEL), particularly in enhancing self-awareness, emotional regulation, empathy demonstration, social skills, and responsible decision-making [179]. This is particularly important in HPE as teaching sensitive topics requires careful planning to ensure effective learning while minimizing strong emotional reactions [180]. Although the application is limited in such areas, one study utilized ChatGPT to improve nursing students’ sexual health knowledge and attitude, including their confidence in managing sexual health-related situations [181]. The result aligned with a recently published pilot study, where teaching using ChatGPT enhanced student confidence in breaking bad news [182], indicating promising capabilities of GenAI in sensitive topic education that necessitates further investigation.

GenAI use in assessment, evaluation, and feedback has shown positive outcomes, including saving time, minimizing cognitive burden on the educator, and decreasing cost compared to a human grader [61, 140]. In addition, GenAI facilitated a more consistent, objective, and timely assessment and feedback of students’ assignments and examinations, thereby permitting the reintroduction of assessment formats that previously were viewed as time- and labor-intensive, such as essay questions [144, 183]. A recent systematic review including educators from different educational level and disciplines, revealed that educators anticipated the profound influence of GenAI on assessment, expressing optimism about its potential to enhance students’ engagement and improve the authenticity of the evaluation process [184]. It further underscored the need to restructure the assessment process in the era of GenAI to more effectively capture critical thinking and higher-order cognitive skills [184]. To address this need, suggested strategies include incorporating AI detection tools, prioritizing the learning process over outcomes, and expanding the use of oral examinations [185]. Another study suggested strategies to design and evaluate the assessment process in the scope of GenAI. In addition, the author highlighted the importance of verifying the assessment validity with the use of multiple GenAI tools to ensure that learning outcomes are not compromised [186]. 

The included studies varied in methodological rigor, encompassing cross-sectional, qualitative, mixed-method, and a limited number of randomized controlled trials [51, 58, 59, 106, 154, 187–194]. The majority of studies relied on subjective self-reported outcomes with limited generalizability due to small sample size, single institution setting, and focus on one discipline. Several studies also exhibited potential biases, including selection, recall, and confounding bias. Furthermore, despite the complexity of the learning process in HPE, only a limited number of studies were grounded in established frameworks or learning theories such as Constructivist Learning Theory, Mayer’s Cognitive Theory, and Technology Acceptance Model (TAM) to inform study design, implementation, and interpretation [119, 173]. Learning theories enhance research quality by offering multiple perspectives to understand the complexity and the associated challenges of HPE learning process [195]. These limitations necessitate a rigorous methodological approach to integrate GenAI into HPE and inform future studies. To guide research conceptualization, a scoping review developed the FACETS framework, a structural approach aimed to enhance the reporting and utility of GenAI findings in medical education [19]. Despite that, a critical appraisal was not conducted, given that scoping reviews are designed to map and explore the available evidence rather than to determine effectiveness [196]. Accordingly, a subsequent systematic review with a focused research question will be necessary to evaluate methodological quality and inform evidence-based recommendations.

The strengths of this scoping review lie in its transparent and rigorous methodology guided by JBI, protocol registration, and adherence to PRISMA-Scr to ensure a systematic approach and minimize reporting bias [30]. Furthermore, a comprehensive search using an iterative approach in seven databases was completed to ensure the inclusion of eligible studies, including grey literature. Unlike previous reviews that highlighted subjectivity in categorizing evidence, this study allowed a single paper to be classified under multiple uses of GenAI, providing a more flexible and accurate representation. Furthermore, by incorporating studies across multiple HPE disciplines, this review provides a deeper and comprehensive understanding of the integration of GenAI across a range of HPE programs that inform and advance further educational changes in such programs. Nonetheless, commentary, editorial, and reviews were excluded, though this is expected to have a minimal impact, as they offered only limited or general insights. Moreover, all eligible primary studies cited by the excluded reviews were identified independently through the search strategy and included if relevant, minimizing the need for secondary resources. Despite these strengths, several limitations are acknowledged. This review included studies published up to January 2025; given the rapidly evolving nature of the topic, more recent studies are not reflected. Furthermore, variations in terminology (e.g., the interchangeable use of GenAI, AI, and chatbot despite their distinction), may have limited the capture of some studies. Additionally, as per the study design, data analysis and quality assessment of the included studies was not performed, which was also reported as a limitation in other scoping reviews [17]. 

Future studies should focus on developing a comprehensive GenAI integration framework that directs effective and safe use. In addition to increasing student and faculty literacy and providing constructive, continuous training on the evolving technology to ensure adequate competencies. Furthermore, it is essential to explore and evaluate the continued integration of different GenAI tools into the education process and to compare its effectiveness. The longer-term impact of GenAI on learning outcomes should be assessed using a longitudinal study design to evaluate its effect on skill development, knowledge retention, and academic performance. Moreover, a comprehensive assessment of the ethical implications of GenAI is essential to inform the development of institutional policies and guidelines that safeguard academic integrity, while evaluating the effectiveness of new AI detection tools and strategies in minimizing plagiarism and preventing academic misconduct.

## Conclusion

This scoping review aimed to synthesize the current literature regarding the integration of generative AI in health professions education. It revealed that GenAI is rapidly being engaged in various educational processes across different disciplines of HPE and within multiple levels of education. Interventional studies indicated its potential in providing personalized learning and transforming traditional education for both students and educators. Students, educators, and administrators generally recognized the benefits and user-friendliness of GenAI, including enhancements in clinical decision-making and knowledge acquisition. To mitigate the risk associated with GenAI integration, institutions are required to proactively develop policies and enhance GenAI literacy among students and educators, while researchers should continuously investigate a broader range of GenAI tools in a more diverse discipline. A clear assessment of integration barriers and facilitators is critical for developing effective and context-specific implementation strategies.

## Supplementary Information

Supplementary Material 1.

Supplementary Material 2

Supplementary Material 3

Supplementary Material 4

Supplementary Material 5

## Data Availability

The data supporting the results of this scoping review are included within the article and in the supplementary materials.

## Abbreviations

HPE: Health profession education
GenAI: Generative artificial intelligence
LLM: Large language model
GPT: Generative pre-trained transformer
ML: Machine learning
DL: Deep learning
NLP: Natural language processing
ANN: Artificial neural network
