# Supplementary material for: A scoping review of the use of generative artificial intelligence tools in health profession education
Source: BMC Med Educ. 2026 Jan 23;26:291. doi: 10.1186/s12909-025-08527-3 (PMC12911342; doi:10.1186/s12909-025-08527-3)
Supplement: Supplementary file 1 — Supplementary Material 1. [file 12909_2025_8527_MOESM1_ESM.docx]

**Supplementary Appendix 1: Figures and Tables**

**Figure S1:** AI subtypes

**Table S1**: AI subtypes definition

| Terminology | Definition |
| --- | --- |
| 1. Traditional AI | |
| Machine Learning (ML) | A computer model/ algorithm that uses given data for prediction and decision-making. The accuracy of the model output and performance increases through the learned data.^(9)^ |
| Artificial Neural Network (ANN) | An essential concept in machine learning that resembles the human nervous system in terms of the data processing strategy. It consists of interconnected nodes that collect and categorize large sets of data using algorithms.^(6)^ |
| Deep Learning (DL) | Another subtype of machine learning. In comparison to ANN, DL has multiple analysis layers, which enable it to process more complex data with minimal chance of error.^(9)^ |
| Natural Language Processing (NLP) | Use of mathematical or computational methods to enable the computers to understand, analyze, and generate responses in natural human language (verbal or written). NLP is usually combined with other AI technology, such in ChatGPT, which consists of NLP and DL.^(10, 11)^ |
| 1. Generative AI (GenAI) | |
| Large Language Model (LLM) | A subset of GenAI that uses DL and NLP to respond to human inquiries and generate a human-like response. LLM is trained on a massive number of natural language documents, AKA corpus, to produce relevant, coherent texts. ^(12, 13)^ |
| Diffusion Model | Another GenAI application is the diffusion model that can generate images from text such as DALL-E.^(7)^ |
