# Supplementary material for: A scoping review of the use of generative artificial intelligence tools in health profession education
Source: BMC Med Educ. 2026 Jan 23;26:291. doi: 10.1186/s12909-025-08527-3 (PMC12911342; doi:10.1186/s12909-025-08527-3)
Supplement: Supplementary file 3 — Supplementary Material 3 [file 12909_2025_8527_MOESM3_ESM.docx]

Search Strategy

# literature search summary

| Databases: PubMed, Scopus, Cochrane Library, ProQuest, CINAHL, ERIC, Embase | |
| --- | --- |
| Population: Health profession education | **Concept: Generative AI** |
| 1. Health profession education 2. Medicine 3. Physician assistant 4. Pharmacy 5. Speech and language therapist 6. Dentistry 7. Nursing 8. Dietician 9. Health science (biomedical, nutrition, physiotherapy, public health) 10. Midwifery 11. Veterinary 12. Paramedic | 1. Generative AI 2. Generative artificial intelligence 3. Gen AI 4. ChatGPT 5. Large language model 6. LLM 7. Generative Pre-Trained Transformer 8. GPT-3 9. GPT-4 |

# Synonyms used

The following table represents the general keywords used in databases. Detailed search strategy is presented in the following section. MeSH terms were used when applicable

| Term | Synonyms |
| --- | --- |
| Generative Artificial intelligence | 1. Generative artificial intelligen* 2. Generative AI 3. Gen AI 4. GenAi 5. ChatGPT 6. Chat GPT 7. Large language model* 8. LLM 9. Generative Pre-Trained Transformer 10. Generative PreTrained Transformer 11. GPT3 12. GPT4 |
| Health profession education | 1. Health profession* educat* 2. Health care profession* educat* 3. Healthcare profession* educat* 4. HPE |
| Medical education | 1. Medic* educat* 2. Medic* student 3. Medic* undergrad* 4. Medic* grad* 5. Medic* college 6. Medic* school*  - If (*) was not recognized by the database both (medical) & (medicine) terms were used with the following terms (education, student, undergraduate, graduate, college, school) |
| Physician associate education | 1. Physician associate*/ physician assist* educat* 2. Physician associate*/ physician assist* student* 3. Physician associate*/ physician assist* undergrad* 4. Physician associate*/ physician assist* grad* 5. Physician associate*/ physician assist* college 6. Physician associate*/ physician assist* school |
| Pharmacy education | 1. pharma* educat* 2. pharma* student* 3. pharma* undergrad* 4. pharma* grad* 5. pharma* college 6. pharma* school  - If (*) was not recognized, both (pharmacy) and (pharma) terms were used. |
| Speech and language therapy education | 1. Speech and language/ slt educat* 2. Speech and language/ slt student* 3. Speech and language/ slt undergrad* 4. Speech and language/ slt grad* 5. Speech and language/ slt college 6. Speech and language/ slt school |
| Nursing education | 1. nurs* educat* 2. nurs* student* 3. nurs* undergrad* 4. nurs* grad* 5. nurs* college 6. nurs* school  - If (*) was not recognized, both (nurse) and (nursing) terms were used. |
| Dentistry education | 1. Dental/ dentistry educat* 2. Dental/ dentistry student* 3. Dental/ dentistry undergrad* 4. Dental/ dentistry grad* 5. Dental/ dentistry college 6. Dental/ dentistry school |
| Health science education | 1. health science* educat* 2. health science* student* 3. health science* undergrad* 4. health science* grad* 5. health science* college 6. health science* school |
| Nutrition education | 1. nutrition educat* 2. nutrition student* 3. nutrition undergrad* 4. nutrition grad* 5. nutrition college 6. nutrition school |
| Dietician education | 1. Dietician/ dietetics educat* 2. Dietician/ dietetics student* 3. Dietician/ dietetics undergrad* 4. Dietician/ dietetics grad* 5. Dietician/ dietetics college 6. Dietician/ dietetics school |
| Biomedical education | 1. biomedic* educat* 2. biomedic* student* 3. biomedic* undergrad* 4. biomedic* grad* 5. biomedic* college 6. biomedic* school  - If (*) was not recognized, both (biomedical) and (biomedicine) terms were used. |
| Physiotherapy education | 1. Physiotherap*/ physical therap*/ occupation* therap* educat* 2. Physiotherap*/ physical therap*/ occupation* therap* student* 3. Physiotherap*/ physical therap*/ occupation* therap* undergrad* 4. Physiotherap*/ physical therap*/ occupation* therap* grad* 5. Physiotherap*/ physical therap*/ occupation* therap* college 6. Physiotherap*/ physical therap*/ occupation* therap* school |
| Public health education | 1. public health educat* 2. public health student* 3. public health undergrad* 4. public health grad* 5. public health college 6. public health school |
| Midwifery education | 1. midwife* educat* 2. midwife* student* 3. midwife* undergrad* 4. midwife* grad* 5. midwife* college 6. midwife* school  - If (*) was not recognized, both (midwife) and (midwifery) terms were used |
| Veterinary education | 1. veterin* educat* 2. veterin* student* 3. veterin* undergrad* 4. veterin* grad* 5. veterin* college 6. veterin* school |
| Paramedic education | 1. paramedic* educat* 2. paramedic* student* 3. paramedic* undergrad* 4. paramedic* grad* 5. paramedic* college 6. paramedic* school |

# Search strategy in databases

### Pubmed

No (*) was used, as more relevant results were identified without applying it.

| #1 | ("generative artificial intelligence" OR "gen ai" OR genai OR "generative ai" OR chatgt OR "chat gpt" OR "large language model" OR "llm" OR "Generative Pre Trained Transformer" OR "Generative PreTrained Transformer" OR “gpt3” OR “gpt4” OR “gpt 3” OR “gpt 4” |
| --- | --- |
| #2 | ("health profession education" OR "health care profession education" OR "HPE") |
| #3 | (medical education OR medical student OR medical undergraduate OR medical graduate OR medical college OR medical school OR medicine education OR medicine student OR medicine undergraduate OR medicine graduate OR medicine college OR medicine school) |
| #4 | (pharmacy education OR pharmacy student OR pharmacy undergraduate OR pharmacy graduate OR pharmacy college OR pharmacy school OR (pharma education OR pharma student OR pharma undergraduate OR pharma graduate OR pharma college OR pharma school) |
| #5 | (nursing education OR nursing student OR nursing undergraduate[ OR nursing graduate OR nursing college OR nursing school OR nurse education OR nurse student OR nurse undergraduate OR nurse graduate OR nurse college OR nurse school) |
| #6 | (dental education OR dental student OR dental undergraduate OR dental graduate OR dental college OR dental school OR dentistry education OR dentistry student OR dentistry undergraduate OR dentistry graduate OR dentistry college OR dentistry school) |
| #7 | ("health science" education) OR ("health science" student) OR ("health science" undergraduate) OR ("health science" graduate) OR ("health science" college) OR ("health science" school) |
| #8 | (nutrition education OR nutrition student OR nutrition undergraduate OR nutrition graduate OR nutrition college OR nutrition school) |
| #9 | (biomedical education OR biomedical student OR biomedical undergraduate OR biomedical graduate OR biomedical college OR biomedical school OR biomedicine education OR biomedicine student OR biomedicine undergraduate OR biomedicine graduate OR biomedicine college OR biomedicine school) |
| #10 | ((physiotherapy education) OR (physiotherapy student) OR (physiotherapy undergraduate) OR (physiotherapy graduate) OR (physiotherapy college) OR (physiotherapy school) OR ("physical therapy" education) OR ("physical therapy" student) OR ("physical therapy" undergraduate) OR ("physical therapy" graduate) OR ("physical therapy" college) OR ("physical therapy" school) OR ("occupation therapy" education) OR ("occupation therapy" student) OR ("occupation therapy" undergraduate) OR ("occupation therapy" graduate) OR ("occupation therapy" college) OR ("occupation therapy" school) |
| #11 | ("public health" education) OR ("public health" student) OR ("public health" undergraduate) OR ("public health" graduate) OR ("public health" college) OR ("public health" school) |
| #12 | (midwifery education OR midwifery student OR midwifery undergraduate OR midwifery graduate OR midwifery college OR midwifery school OR midwife education OR midwife student OR midwife undergraduate OR midwife graduate OR midwife college OR midwife school) |
| #13 | (veterinary education OR veterinary student OR veterinary undergraduate OR veterinary graduate OR veterinary college OR veterinary school) |
| #14 | (paramedic education OR paramedic student OR paramedic undergraduate OR paramedic graduate OR paramedic college OR paramedic school OR paramedicine education OR paramedicine student OR paramedicine undergraduate OR paramedicine graduate OR paramedicine college OR paramedicine school) |
| #15 | (dietician education OR dietician student OR dietician undergraduate OR dietician graduate OR dietician college OR dietician school OR dietetics education OR dietetics student OR dietetics undergraduate OR dietetics graduate OR dietetics college OR dietetics school) |
| #16 | (physician assistant education OR physician assistant student OR physician assistant undergraduate OR physician assistant graduate OR physician assistant college OR physician assistant school OR physician associate education OR physician associate student OR physician associate undergraduate OR physician associate graduate OR physician associate college OR physician associate school) |
| #17 | ("speech and language" education OR "speech and language" student OR "speech and language" undergraduate OR "speech and language" graduate OR "speech and language" school OR "speech and language" college OR "slt" education OR "slt" student OR "slt" undergraduate OR "slt" graduate OR "slt" college OR "slt" school) |
| #18 | #2 OR #3 OR #4 OR #5 OR #6 OR #7 OR #8 OR #9 OR #10 OR #11 OR #12 OR #13 OR #14 OR #15 OR #16 OR #17 |
| #19 | #1 AND #18 |

### Embase, Scopus, ProQuest Central, CINAHL and ERIC

| #1 | ("generative artificial intelligen*" OR "gen ai" OR genai OR “generative ai” OR chatgpt OR "chat gpt" OR "large language model*" OR "llm" OR "Generative Pre Trained Transformer" OR "Generative PreTrained Transformer" OR “gpt3” OR “gpt 3” OR “gpt4” OR “gpt4” |
| --- | --- |
| #2 | ( "health profession* educat*" ) OR ( "health care profession* educat*" ) OR ( "healthcare profession* educat*" ) OR ( "HPE" ) |
| #3 | ( medic* educat* ) OR ( medic* student ) OR ( medic* undergrad* ) OR ( medic* grad* ) OR ( medic* college ) OR ( medic* school ) |
| #4 | (pharma* grad* ) OR ( pharma* college ) OR ( pharma* school ) |
| #5 | ( nurs* educat* ) OR ( nurs* student* ) OR ( nurs* undergrad* ) OR ( nurs* grad* ) OR ( nurs* college ) OR ( nurs* school ) |
| #6 | ( dental educat* ) OR ( dental student* ) OR ( dental undergrad* ) OR ( dental grad* ) OR ( dental college ) OR ( dental school ) OR ( dentist* educat* ) OR ( dentist* student* ) OR ( dentist* undergrad* ) OR ( dentist* grad* ) OR ( dentist* college ) OR ( dentist* school ) |
| #7 | ( "health science*" educat* ) OR ( "health science*" student* ) OR ( "health science*" undergrad* ) OR ( "health science*" grad* ) OR ( "health science*" college ) OR ( "health science*" school ) |
| #8 | ( nutrition educat* ) OR ( nutrition student* ) OR ( nutrition undergrad* ) OR ( nutrition grad* ) OR ( nutrition college ) OR ( nutrition school ) |
| #9 | ( biomedic* educat* ) OR ( biomedic* student* ) OR ( biomedic* undergrad* ) OR ( biomedic* grad* ) OR ( biomedic* college ) OR ( biomedic* school ) |
| #10 | ( physiotherap* educat* ) OR ( physiotherap* student* ) OR ( physiotherap* undergrad* ) OR ( physiotherap* grad* ) OR ( physiotherap* college ) OR ( physiotherap* school ) OR ( "physical therap*" educat* ) OR ( "physical therap*" student* ) OR ( "physical therap*" undergrad* ) OR ( "physical therap*" grad* ) OR ( "physical therap*" college ) OR ( "physical therap*" school ) OR ( "occupation* therap*" educat* ) OR ( "occupation* therap*" student* ) OR ( "occupation* therap*" undergrad* ) OR ( "occupation* therap*" grad* ) OR ( "occupation* therap*" college ) OR ( "occupation* therap*" school ) |
| #11 | ( "public health" educat* ) OR ( "public health" student* ) OR ( "public health" undergrad* ) OR ( "public health" grad* ) OR ( "public health" college ) OR ( "public health" school ) |
| #12 | ( midwife* educat* ) OR ( midwife* student* ) OR ( midwife* undergrad* ) OR ( midwife* grad* ) OR ( midwife* college ) OR ( midwife* school ) |
| #13 | ( veterin* educat* ) OR ( veterin* student* ) OR ( veterin* undergrad* ) OR ( veterin* grad* ) OR ( veterin* college ) OR ( veterin* school ) |
| #14 | ( paramedic* educat* ) OR ( paramedic* student* ) OR ( paramedic* undergrad* ) OR ( paramedic* grad* ) OR ( paramedic* college ) OR ( paramedic* school ) |
| #15 | OR ( "speech and language" educat* ) OR ( "speech and language" student* ) OR ( "speech and language" undergrad* ) OR ( "speech and language" grad* ) OR ( "speech and language" college ) OR ( "speech and language" school ) OR ( "slt" educat* ) OR ( "slt" student* ) OR ( "slt" undergrad* ) OR ( "slt" grad* ) OR ( "slt" college ) OR ( "slt" school ) |
| #16 | ( "physician assist*" educat* ) OR ( "physician assist*" student* ) OR ( "physician assist*" undergrad* ) OR ( "physician assist*" grad* ) OR ( "physician assist*" college ) OR ( "physician assist*" school ) OR ( "physician associate*" educat* ) OR ( "physician associate*" student* ) OR ( "physician associate*" undergrad* ) OR ( "physician associate*" grad* ) OR ( "physician associate*" college ) OR ( "physician associate*" school ) |
| #17 | ( dietician educat* ) OR ( dietician student* ) OR ( dietician undergrad* ) OR ( dietician grad* ) OR ( dietician college ) OR ( dietician school ) OR ( dietetic educat* ) OR ( dietetic student* ) OR ( dietetic undergrad* ) OR ( dietetic grad* ) OR ( dietetic college ) OR ( dietetic school |
| #18 | #2 OR #3 OR #4 OR #5 OR #6 OR #7 OR #8 OR #9 OR #10 OR #11 OR #12 OR #13 OR #14 OR #15 OR #16 OR #17 |
| #19 | #1 AND #18 |

### Cochrane Library

| #1 | ((generative NEXT artificial NEXT intelligen*) OR (gen NEXT ai) OR (genai) OR (generative NEXT ai) OR (chatgpt) OR (chat NEXT gpt) OR (large NEXT language NEXT model*) OR "llm" OR (Generative NEXT Pre NEXT Trained NEXT Transformer) OR (Generative NEXT PreTrained NEXT Transformer) OR “gpt3” OR “gpt 3” OR “gpt4” OR “gpt 4”) |
| --- | --- |
| #2 | MeSH descriptor: [Students, Health Occupation] explode all trees |
| #3 | MeSH descriptor: [Schools, Health Occupations] explode all trees |
| #4 | MeSH descriptor: [Education, Professional] explode all trees |
| #5 | MeSH descriptor: [Education, Graduate] explode all trees |
| #6 | ((health NEXT profession* NEXT educat*) OR (healthcare NEXT profession* NEXT educat*) OR (health NEXT care NEXT profession* NEXT educat*) OR (HPE) OR ((health NEXT profession*) student*) OR ((health NEXT profession*) undergrad*) OR ((health NEXT profession*) grad*) OR ((health NEXT profession*) college) OR ((health NEXT profession*) school)):ti,ab,kw |
| #7 | (medic* student*) OR (medic* educat*) OR (medic* undergrad*) OR (medic* grad*) OR (medic* college*) OR (medic* school) |
| #8 | (pharma* student*)OR (pharma* educat*) OR (pharma* undergrad*) OR (pharma* grad*) OR (pharma* college) OR (pharma* school) |
| #9 | (nurs* student*) OR (nurs* educat*) OR (nurs* undergrad*) OR (nurs* grad*) OR (nurs* college) OR (nurs* school) |
| #10 | (dental student*) OR (dental educat*) OR (dental undergrad*) OR (dental grad*) OR (dental college) OR (dental school) OR (dentist* student*) OR (dentist* educat*) OR (dentist* undergrad*) OR (dentist* grad*) OR (dentist* college) OR (dentist* school) |
| #11 | (dietician AND educat*) OR (dietician AND student*) OR (dietician AND undergrad*) OR (dietician AND grad*) OR (dietician AND college) OR (dietician AND school) OR (dietetics AND educat*) OR (dietetics AND student*) OR (dietetics AND undergrad*) OR (dietetics AND grad*) OR (dietetics AND college) OR (dietetics AND school) |
| #12 | (physician NEXT assist*) AND educat*) OR ((physician NEXT assist*) AND student*) OR ((physician NEXT assist*) AND undergrad*) OR ((physician NEXT assist*) AND grad*) OR ((physician NEXT assist*) AND college) OR ((physician NEXT assist*) AND school) OR ((physician NEXT associate*) AND educat*) OR ((physician NEXT associate*) AND student*) OR ((physician NEXT associate*) AND undergrad*) OR ((physician NEXT associate*) AND grad*) OR ((physician NEXT associate*) AND college) OR ((physician NEXT associate*) AND school) |
| #13 | (health NEXT science*) student*) OR ((health NEXT science*) educat*) OR ((health NEXT science*) undergrad*) OR ((health NEXT science*) grad*) OR ((health NEXT science*) college) OR ((health NEXT science*) school) |
| #14 | (nutrition student*) OR (nutrition educat*) OR (nutrition college) OR (nutrition undergrad*) OR (nutrition grad*) OR (nutrition school) |
| #15 | ((physical NEXT therap*) student*) OR ((physical NEXT therap*) undergrad*) OR ((physical NEXT therap*) grad*) OR ((physical NEXT therap*) educat*) OR ((physical NEXT therap*) college) OR ((physical NEXT therap*) school)) |
| #16 | ((occupation* NEXT therap*) student*) OR ((occupation* NEXT therap*) undergrad*) OR ((occupation* NEXT therap*) grad*) OR ((occupation* NEXT therap*) educat*) OR ((occupation* NEXT therap*) college) OR ((occupation* NEXT therap*) school)) |
| #17 | (physiotherap* student*) OR (physiotherap* undergrad*) OR (physiotherap* grad*) OR (physiotherap* educat*) OR (physiotherap* college) OR (physiotherap* school) |
| #18 | ((public NEXT health) student*) OR ((public NEXT health) educat*) OR ((public NEXT health) undergrad*) OR ((public NEXT health) grad*) OR ((public NEXT health) college) OR ((public NEXT health) school) |
| #19 | (biomedic* student*) OR (biomedic* undergrad*) OR (biomedic* grad*) OR (biomedic* educat*) OR (biomedic* college) OR (biomedic* school) |
| #20 | ((veterin* student*) OR (veterin* educat*) OR (veterin* undergrad*) OR (veterin* grad*) OR (veterin* college) OR (veterin* school)):ti,ab,kw |
| #21 | (midwife* student*) OR (midwife* educat*) OR (midwife* undergrad*) OR (midwife* grad*) OR (midwife* college) OR (midwife* school) |
| #22 | (paramedic* student*) OR (paramedic* undergrad*) OR (paramedic* grad*) OR (paramedic* college) OR (paramedic* school) |
| #23 | (((speech NEAR language) educat*) OR ((speech NEAR language) student*) OR ((speech NEAR language) undergrad*) OR ((speech NEAR language) grad*) OR ((speech NEAR language) college) OR ((speech NEAR language) school) OR (“slt” AND educat*) OR (“slt” AND student*) OR (“slt” AND undergad*) OR (“slt” AND grad*) OR (“slt” AND college) OR (“slt” AND school) |
| #24 | {OR #2-23} |
| #25 | #1 AND #24 |

# Limitation applied

| PubMed | No limitation was applied (search was conducted in All Field) |
| --- | --- |
| Embase | Limit the search to title, abstract and explosion (ti,ab,exp) |
| Scopus | Limit the search to title, abstract, and keywords |
| Cochrane Library | Limit the search to title, abstract and keywords |
| ProQuest | Limit the search to “anywhere except full text (NOFT)”   - **Include:**   scholarly journal, conference paper, and proceedings, working paper, other resource, dissertation and thesis, and reports   - Exclude:   Trade journal, wire feeds, newspapers, blogs, podcasts, websites, magazines, audio and video works. |

# Number of studies identified from databases

| CINAHL | 311 |
| --- | --- |
| Cochrane Library | 48 |
| Embase | 3447 |
| ERIC | 29 |
| ProQuest | 2913 |
| PubMed | 4823 |
| Scopus | 2637 |
| Total: 14,208 | |

- Total number is based on a search conducted on 31 Jan 2025.

# Combined search strategy (Query)

### PubMed

| ((((((((((((((((("public health" education) OR ("public health" student) OR ("public health" undergraduate) OR ("public health" graduate) OR ("public health" college) OR ("public health" school)) OR (paramedic education OR paramedic student OR paramedic undergraduate OR paramedic graduate OR paramedic college OR paramedic school)) OR (paramedicine education OR paramedicine student OR paramedicine undergraduate OR paramedicine graduate OR paramedicine college OR paramedicine school))OR (veterinary education OR veterinary student OR veterinary undergraduate OR veterinary graduate OR veterinary college OR veterinary school)) OR (midwifery education OR midwifery student OR midwifery undergraduate OR midwifery graduate OR midwifery college OR midwifery school)) OR (midwife education OR midwife student OR midwife undergraduate OR midwife graduate OR midwife college OR midwife school)) OR ((physiotherapy education) OR (physiotherapy student) OR (physiotherapy undergraduate) OR (physiotherapy graduate) OR (physiotherapy college) OR (physiotherapy school) OR ("physical therapy" education) OR ("physical therapy" student) OR ("physical therapy" undergraduate) OR ("physical therapy" graduate) OR ("physical therapy" college) OR ("physical therapy" school) OR ("occupation therapy" education) OR ("occupation therapy" student) OR ("occupation therapy" undergraduate) OR ("occupation therapy" graduate) OR ("occupation therapy") college OR ("occupation therapy" school))) OR (biomedical education OR biomedical student OR biomedical undergraduate OR biomedical graduate OR biomedical college OR biomedical school OR biomedicine education OR biomedicine student OR biomedicine undergraduate OR biomedicine graduate OR biomedicine college OR biomedicine school)) OR (nutrition education OR nutrition student OR nutrition undergraduate OR nutrition graduate OR nutrition college OR nutrition school)) OR (("health science" education) OR ("health science" student) OR ("health science" undergraduate) OR ("health science" graduate) OR ("health science" college) OR ("health science" school))) OR (dental education OR dental student OR dental undergraduate OR dental graduate OR dental college OR dental school OR dentistry education OR dentistry student OR dentistry undergraduate OR dentistry graduate OR dentistry college OR dentistry school)) OR (nursing education OR nursing student OR nursing undergraduate OR nursing graduate OR nursing college OR nursing school OR nurse education OR nurse student OR nurse undergraduate OR nurse graduate OR nurse college OR nurse school)) OR (pharmacy education OR pharmacy student OR pharmacy undergraduate OR pharmacy graduate OR pharmacy college OR pharmacy school)) OR (medicine education OR medicine student OR medicine undergraduate OR medicine graduate OR medicine college OR medicine school)) OR (pharma education OR pharma student OR pharma undergraduate OR pharma graduate OR pharma college OR pharma school)) OR (medical education OR medical student OR medical undergraduate OR medical graduate OR medical college OR medical school)) OR ("health profession education" OR "health care profession education" OR "HPE") OR (dietician education OR dietician student OR dietician undergraduate OR dietician graduate OR dietician college OR dietician school) OR (dietetics education OR dietetics student OR dietetics undergraduate OR dietetics graduate OR dietetics college OR dietetics school) OR (physician assistant education OR physician assistant student* OR physician assistant undergraduate OR physician assistant graduate OR physician assistant college OR physician assistant school) OR (physician associate education OR physician associate student OR physician associate undergraduate OR physician associate graduate OR physician associate college OR physician associate school) OR ("speech and language" education OR "speech and language" student OR "speech and language" undergraduate OR "speech and language" graduate OR "speech and language" school OR "speech and language" college) OR ("slt" education OR "slt" student OR "slt" undergraduate OR "slt" graduate OR "slt" college OR "slt" school)) AND ("Generative PreTrained Transformer" OR "Generative Pre Trained Transformer" OR "llm" OR "large language model" OR "chat gpt" OR "chatgpt" OR "generative ai" OR "gen ai" OR "genai" OR "generative artificial intelligence" OR "gpt3" OR "gpt4" OR "gpt 3" OR "gpt 4") |
| --- |

### EMBASE

| ('generative artificial intelligen*':ab,ti,exp OR 'gen ai':ab,ti,exp OR genai:ab,ti,exp OR 'generative ai':ab,ti,exp OR chatgpt:ab,ti,exp OR 'chat gpt':ab,ti,exp OR 'large language model*':ab,ti,exp OR 'llm':ab,ti,exp OR 'generative pre trained transformer':ab,ti,exp OR 'generative pretrained transformer':ab,ti,exp OR 'gpt3':ab,ti,exp OR 'gpt4':ab,ti,exp OR 'gpt 3':ab,ti,exp OR 'gpt 4':ab,ti,exp) AND ('health profession* and educat*':ab,ti,exp OR 'health care profession* and educat*':ab,ti,exp OR 'healthcare profession* and educat*':ab,ti,exp OR 'hpe':ab,ti,exp OR (medic*:ab,ti,exp AND educat*:ab,ti,exp) OR (medic*:ab,ti,exp AND student:ab,ti,exp) OR (medic*:ab,ti,exp AND undergrad*:ab,ti,exp) OR (medic*:ab,ti,exp AND grad*:ab,ti,exp) OR (medic*:ab,ti,exp AND college:ab,ti,exp) OR (medic*:ab,ti,exp AND school*:ab,ti,exp) OR (pharma*:ab,ti,exp AND educat*:ab,ti,exp) OR (pharma*:ab,ti,exp AND student*:ab,ti,exp) OR (pharma*:ab,ti,exp AND undergrad*:ab,ti,exp) OR (pharma*:ab,ti,exp AND grad*:ab,ti,exp) OR (pharma*:ab,ti,exp AND college:ab,ti,exp) OR (pharma*:ab,ti,exp AND school:ab,ti,exp) OR (nurs*:ab,ti,exp AND educat*:ab,ti,exp) OR (nurs*:ab,ti,exp AND student*:ab,ti,exp) OR (nurs*:ab,ti,exp AND undergrad*:ab,ti,exp) OR (nurs*:ab,ti,exp AND grad*:ab,ti,exp) OR (nurs*:ab,ti,exp AND college:ab,ti,exp) OR (nurs*:ab,ti,exp AND school:ab,ti,exp) OR (dental:ab,ti,exp AND educat*:ab,ti,exp) OR (dental:ab,ti,exp AND student*:ab,ti,exp) OR (dental:ab,ti,exp AND undergrad*:ab,ti,exp) OR (dental:ab,ti,exp AND grad*:ab,ti,exp) OR (dental:ab,ti,exp AND college:ab,ti,exp) OR (dental:ab,ti,exp AND school:ab,ti,exp) OR (dentist*:ab,ti,exp AND educat*:ab,ti,exp) OR (dentist*:ab,ti,exp AND student*:ab,ti,exp) OR (dentist*:ab,ti,exp AND undergrad*:ab,ti,exp) OR (dentist*:ab,ti,exp AND grad*:ab,ti,exp) OR (dentist*:ab,ti,exp AND college:ab,ti,exp) OR (dentist*:ab,ti,exp AND school:ab,ti,exp) OR ('health science*':ab,ti,exp AND educat*:ab,ti,exp) OR ('health science*':ab,ti,exp AND student*:ab,ti,exp) OR ('health science*':ab,ti,exp AND undergrad*:ab,ti,exp) OR ('health science*':ab,ti,exp AND grad*:ab,ti,exp) OR ('health science*':ab,ti,exp AND college:ab,ti,exp) OR ('health science*':ab,ti,exp AND school:ab,ti,exp) OR (nutrition:ab,ti,exp AND educat*:ab,ti,exp) OR (nutrition:ab,ti,exp AND student*:ab,ti,exp) OR (nutrition:ab,ti,exp AND undergrad*:ab,ti,exp) OR (nutrition:ab,ti,exp AND grad*:ab,ti,exp) OR (nutrition:ab,ti,exp AND college:ab,ti,exp) OR (nutrition:ab,ti,exp AND school:ab,ti,exp) OR (biomedic*:ab,ti,exp AND educat*:ab,ti,exp) OR (biomedic*:ab,ti,exp AND student*:ab,ti,exp) OR (biomedic*:ab,ti,exp AND undergrad*:ab,ti,exp) OR (biomedic*:ab,ti,exp AND grad*:ab,ti,exp) OR (biomedic*:ab,ti,exp AND college:ab,ti,exp) OR (biomedic*:ab,ti,exp AND school:ab,ti,exp) OR (physiotherap*:ab,ti,exp AND educat*:ab,ti,exp) OR (physiotherap*:ab,ti,exp AND student*:ab,ti,exp) OR (physiotherap*:ab,ti,exp AND undergrad*:ab,ti,exp) OR (physiotherap*:ab,ti,exp AND grad*:ab,ti,exp) OR (physiotherap*:ab,ti,exp AND college:ab,ti,exp) OR (physiotherap*:ab,ti,exp AND school:ab,ti,exp) OR ('physical therap*':ab,ti,exp AND educat*:ab,ti,exp) OR ('physical therap*':ab,ti,exp AND student*:ab,ti,exp) OR ('physical therap*':ab,ti,exp AND undergrad*:ab,ti,exp) OR ('physical therap*':ab,ti,exp AND grad*:ab,ti,exp) OR ('physical therap*':ab,ti,exp AND college:ab,ti,exp) OR ('physical therap*':ab,ti,exp AND school:ab,ti,exp) OR ('occupation* therap*':ab,ti,exp AND educat*:ab,ti,exp) OR ('occupation* therap*':ab,ti,exp AND student*:ab,ti,exp) OR ('occupation* therap*':ab,ti,exp AND undergrad*:ab,ti,exp) OR ('occupation* therap*':ab,ti,exp AND grad*:ab,ti,exp) OR ('occupation* therap*':ab,ti,exp AND college:ab,ti,exp) OR ('occupation* therap*':ab,ti,exp AND school:ab,ti,exp) OR ('public health':ab,ti,exp AND educat*:ab,ti,exp) OR ('public health':ab,ti,exp AND student*:ab,ti,exp) OR ('public health':ab,ti,exp AND undergrad*:ab,ti,exp) OR ('public health':ab,ti,exp AND grad*:ab,ti,exp) OR ('public health':ab,ti,exp AND college:ab,ti,exp) OR ('public health':ab,ti,exp AND school:ab,ti,exp) OR (midwife*:ab,ti,exp AND educat*:ab,ti,exp) OR (midwife*:ab,ti,exp AND student*:ab,ti,exp) OR (midwife*:ab,ti,exp AND undergrad*:ab,ti,exp) OR (midwife*:ab,ti,exp AND grad*:ab,ti,exp) OR (midwife*:ab,ti,exp AND college:ab,ti,exp) OR (midwife*:ab,ti,exp AND school:ab,ti,exp) OR (veterin*:ab,ti,exp AND educat*:ab,ti,exp) OR (veterin*:ab,ti,exp AND student*:ab,ti,exp) OR (veterin*:ab,ti,exp AND undergrad*:ab,ti,exp) OR (veterin*:ab,ti,exp AND grad*:ab,ti,exp) OR (veterin*:ab,ti,exp AND college:ab,ti,exp) OR (veterin*:ab,ti,exp AND school:ab,ti,exp) OR (paramedic*:ab,ti,exp AND educat*:ab,ti,exp) OR (paramedic*:ab,ti,exp AND student*:ab,ti,exp) OR (paramedic*:ab,ti,exp AND undergrad*:ab,ti,exp) OR (paramedic*:ab,ti,exp AND grad*:ab,ti,exp) OR (paramedic*:ab,ti,exp AND college:ab,ti,exp) OR (paramedic*:ab,ti,exp AND school:ab,ti,exp) OR ('speech and language':ab,ti,exp AND educat*:ab,ti,exp) OR ('speech and language':ab,ti,exp AND student*:ab,ti,exp) OR ('speech and language':ab,ti,exp AND undergrad*:ab,ti,exp) OR ('speech and language':ab,ti,exp AND grad*:ab,ti,exp) OR ('speech and language':ab,ti,exp AND college:ab,ti,exp) OR ('speech and language':ab,ti,exp AND school:ab,ti,exp) OR ('slt':ab,ti,exp AND educat*:ab,ti,exp) OR ('slt':ab,ti,exp AND student*:ab,ti,exp) OR ('slt':ab,ti,exp AND undergrad*:ab,ti,exp) OR ('slt':ab,ti,exp AND grad*:ab,ti,exp) OR ('slt':ab,ti,exp AND college:ab,ti,exp) OR ('slt':ab,ti,exp AND school:ab,ti,exp) OR ('physician assist*':ab,ti,exp AND educat*:ab,ti,exp) OR ('physician assist*':ab,ti,exp AND student*:ab,ti,exp) OR ('physician assist*':ab,ti,exp AND undergrad*:ab,ti,exp) OR ('physician assist*':ab,ti,exp AND grad*:ab,ti,exp) OR ('physician assist*':ab,ti,exp AND college:ab,ti,exp) OR ('physician assist*':ab,ti,exp AND school:ab,ti,exp) OR ('physician associate*':ab,ti,exp AND educat*:ab,ti,exp) OR ('physician associate*':ab,ti,exp AND student*:ab,ti,exp) OR ('physician associate*':ab,ti,exp AND undergrad*:ab,ti,exp) OR ('physician associate*':ab,ti,exp AND grad*:ab,ti,exp) OR ('physician associate*':ab,ti,exp AND college:ab,ti,exp) OR ('physician associate*':ab,ti,exp AND school:ab,ti,exp) OR (dietician:ab,ti,exp AND educat*:ab,ti,exp) OR (dietician:ab,ti,exp AND student*:ab,ti,exp) OR (dietician:ab,ti,exp AND undergrad*:ab,ti,exp) OR (dietician:ab,ti,exp AND grad*:ab,ti,exp) OR (dietician:ab,ti,exp AND college:ab,ti,exp) OR (dietician:ab,ti,exp AND school:ab,ti,exp) OR (dietetics:ab,ti,exp AND educat*:ab,ti,exp) OR (dietetics:ab,ti,exp AND student*:ab,ti,exp) OR (dietetics:ab,ti,exp AND undergrad*:ab,ti,exp) OR (dietetics:ab,ti,exp AND grad*:ab,ti,exp) OR (dietetics:ab,ti,exp AND college:ab,ti,exp) OR (dietetics:ab,ti,exp AND school:ab,ti,exp)) AND (2018:py OR 2019:py OR 2020:py OR 2021:py OR 2022:py OR 2023:py OR 2024:py OR 2025:py) |
| --- |

### CINAHL

| (( ( biomedic* educat* ) OR ( biomedic* student* ) OR ( biomedic* undergrad* ) OR ( biomedic* grad* ) OR ( biomedic* college ) OR ( biomedic* school ) OR ( "health profession* educat*" ) OR ( "health care profession* educat*" ) OR ( "healthcare profession* educat*" ) OR ( "HPE" ) OR ( medic* educat* ) OR ( medic* student ) OR ( medic* undergrad* ) OR ( medic* grad* ) OR ( medic* college ) OR ( medic* school ) OR ( pharma* educat* ) OR ( pharma* student* ) OR ( pharma* undergrad* ) OR ( pharma* grad* ) OR ( pharma* college ) OR ( pharma* school ) OR ( nurs* educat* ) OR ( nurs* student* ) OR ( nurs* undergrad* ) OR ( nurs* grad* ) OR ( nurs* college ) OR ( nurs* school ) OR ( dental educat* ) OR ( dental student* ) OR ( dental undergrad* ) OR ( dental grad* ) OR ( dental college ) OR ( dental school ) OR ( dentist* educat* ) OR ( dentist* student* ) OR ( dentist* undergrad* ) OR ( dentist* grad* ) OR ( dentist* college ) OR ( dentist* school ) OR ( "health science*" educat* ) OR ( "health science*" student* ) OR ( "health science*" undergrad* ) OR ( "health science*" grad* ) OR ( "health science*" college ) OR ( "health science*" school ) OR ( nutrition educat* ) OR ( nutrition student* ) OR ( nutrition undergrad* ) OR ( nutrition grad* ) OR ( nutrition college ) OR ( nutrition school ) OR ( physiotherap* educat* ) OR ( physiotherap* student* ) OR ( physiotherap* undergrad* ) OR ( physiotherap* grad* ) OR ( physiotherap* college ) OR ( physiotherap* school ) OR ( "physical therap*" educat* ) OR ( "physical therap*" student* ) OR ( "physical therap*" undergrad* ) OR ( "physical therap*" grad* ) OR ( "physical therap*" college ) OR ( "physical therap*" school ) OR ( "occupation* therap*" educat* ) OR ( "occupation* therap*" student* ) OR ( "occupation* therap*" undergrad* ) OR ( "occupation* therap*" grad* ) OR ( "occupation* therap*" college ) OR ( "occupation* therap*" school ) OR ( "public health" educat* ) OR ( "public health" student* ) OR ( "public health" undergrad* ) OR ( "public health" grad* ) OR ( "public health" college ) OR ( "public health" school ) OR ( midwife* educat* ) OR ( midwife* student* ) OR ( midwife* undergrad* ) OR ( midwife* grad* ) OR ( midwife* college ) OR ( midwife* school ) OR ( veterin* educat* ) OR ( veterin* student* ) OR ( veterin* undergrad* ) OR ( veterin* grad* ) OR ( veterin* college ) OR ( veterin* school ) OR ( paramedic* educat* ) OR ( paramedic* student* ) OR ( paramedic* undergrad* ) OR ( paramedic* grad* ) OR ( paramedic* college ) OR ( paramedic* school ) OR ( "speech and language" educat* ) OR ( "speech and language" student* ) OR ( "speech and language" undergrad* ) OR ( "speech and language" grad* ) OR ( "speech and language" college ) OR ( "speech and language" school ) OR ( "slt" educat* ) OR ( "slt" student* ) OR ( "slt" undergrad* ) OR ( "slt" grad* ) OR ( "slt" college ) OR ( "slt" school ) OR ( "physician assist*" educat* ) OR ( "physician assist*" student* ) OR ( "physician assist*" undergrad* ) OR ( "physician assist*" grad* ) OR ( "physician assist*" college ) OR ( "physician assist*" school ) OR ( "physician associate*" educat* ) OR ( "physician associate*" student* ) OR ( "physician associate*" undergrad* ) OR ( "physician associate*" grad* ) OR ( "physician associate*" college ) OR ( "physician associate*" school ) OR ( dietician educat* ) OR ( dietician student* ) OR ( dietician undergrad* ) OR ( dietician grad* ) OR ( dietician college ) OR ( dietician school ) OR ( dietetic educat* ) OR ( dietetic student* ) OR ( dietetic undergrad* ) OR ( dietetic grad* ) OR ( dietetic college ) OR ( dietetic school)) AND (( "generative artificial intelligen*" ) OR ( "gen ai" ) OR ( genai ) OR ( "generative ai" ) OR ( chatgpt ) OR ( "chat gpt" ) OR ( "large language model*" ) OR ( "llm" ) OR ( "Generative Pre Trained Transformer" ) OR ( "Generative PreTrained Transformer" ) OR ( gpt3 ) OR ( gpt4 ) OR ( gpt 3 ) OR ( gpt 4 )) |
| --- |

### ERIC

| (( ( biomedic* educat* ) OR ( biomedic* student* ) OR ( biomedic* undergrad* ) OR ( biomedic* grad* ) OR ( biomedic* college ) OR ( biomedic* school ) OR ( "health profession* educat*" ) OR ( "health care profession* educat*" ) OR ( "healthcare profession* educat*" ) OR ( "HPE" ) OR ( medic* educat* ) OR ( medic* student ) OR ( medic* undergrad* ) OR ( medic* grad* ) OR ( medic* college ) OR ( medic* school ) OR ( pharma* educat* ) OR ( pharma* student* ) OR ( pharma* undergrad* ) OR ( pharma* grad* ) OR ( pharma* college ) OR ( pharma* school ) OR ( nurs* educat* ) OR ( nurs* student* ) OR ( nurs* undergrad* ) OR ( nurs* grad* ) OR ( nurs* college ) OR ( nurs* school ) OR ( dental educat* ) OR ( dental student* ) OR ( dental undergrad* ) OR ( dental grad* ) OR ( dental college ) OR ( dental school ) OR ( dentist* educat* ) OR ( dentist* student* ) OR ( dentist* undergrad* ) OR ( dentist* grad* ) OR ( dentist* college ) OR ( dentist* school ) OR ( "health science*" educat* ) OR ( "health science*" student* ) OR ( "health science*" undergrad* ) OR ( "health science*" grad* ) OR ( "health science*" college ) OR ( "health science*" school ) OR ( nutrition educat* ) OR ( nutrition student* ) OR ( nutrition undergrad* ) OR ( nutrition grad* ) OR ( nutrition college ) OR ( nutrition school ) OR ( physiotherap* educat* ) OR ( physiotherap* student* ) OR ( physiotherap* undergrad* ) OR ( physiotherap* grad* ) OR ( physiotherap* college ) OR ( physiotherap* school ) OR ( "physical therap*" educat* ) OR ( "physical therap*" student* ) OR ( "physical therap*" undergrad* ) OR ( "physical therap*" grad* ) OR ( "physical therap*" college ) OR ( "physical therap*" school ) OR ( "occupation* therap*" educat* ) OR ( "occupation* therap*" student* ) OR ( "occupation* therap*" undergrad* ) OR ( "occupation* therap*" grad* ) OR ( "occupation* therap*" college ) OR ( "occupation* therap*" school ) OR ( "public health" educat* ) OR ( "public health" student* ) OR ( "public health" undergrad* ) OR ( "public health" grad* ) OR ( "public health" college ) OR ( "public health" school ) OR ( midwife* educat* ) OR ( midwife* student* ) OR ( midwife* undergrad* ) OR ( midwife* grad* ) OR ( midwife* college ) OR ( midwife* school ) OR ( veterin* educat* ) OR ( veterin* student* ) OR ( veterin* undergrad* ) OR ( veterin* grad* ) OR ( veterin* college ) OR ( veterin* school ) OR ( paramedic* educat* ) OR ( paramedic* student* ) OR ( paramedic* undergrad* ) OR ( paramedic* grad* ) OR ( paramedic* college ) OR ( paramedic* school ) OR ( "speech and language" educat* ) OR ( "speech and language" student* ) OR ( "speech and language" undergrad* ) OR ( "speech and language" grad* ) OR ( "speech and language" college ) OR ( "speech and language" school ) OR ( "slt" educat* ) OR ( "slt" student* ) OR ( "slt" undergrad* ) OR ( "slt" grad* ) OR ( "slt" college ) OR ( "slt" school ) OR ( "physician assist*" educat* ) OR ( "physician assist*" student* ) OR ( "physician assist*" undergrad* ) OR ( "physician assist*" grad* ) OR ( "physician assist*" college ) OR ( "physician assist*" school ) OR ( "physician associate*" educat* ) OR ( "physician associate*" student* ) OR ( "physician associate*" undergrad* ) OR ( "physician associate*" grad* ) OR ( "physician associate*" college ) OR ( "physician associate*" school ) OR ( dietician educat* ) OR ( dietician student* ) OR ( dietician undergrad* ) OR ( dietician grad* ) OR ( dietician college ) OR ( dietician school ) OR ( dietetic educat* ) OR ( dietetic student* ) OR ( dietetic undergrad* ) OR ( dietetic grad* ) OR ( dietetic college ) OR ( dietetic school)) AND (( "generative artificial intelligen*" ) OR ( "gen ai" ) OR ( genai ) OR ( "generative ai" ) OR ( chatgpt ) OR ( "chat gpt" ) OR ( "large language model*" ) OR ( "llm" ) OR ( "Generative Pre Trained Transformer" ) OR ( "Generative PreTrained Transformer" ) OR ( gpt3 ) OR ( gpt4 ) OR ( gpt 3 ) OR ( gpt 4 )) |
| --- |

### Scopus

| ( TITLE-ABS-KEY ( ( biomedic* AND educat* ) OR ( biomedic* AND student* ) OR ( biomedic* AND undergrad* ) OR ( biomedic* AND grad* ) OR ( biomedic* AND college ) OR ( biomedic* AND school ) OR ( "health profession* educat*" ) OR ( "health care profession* educat*" ) OR ( "healthcare profession* educat*" ) OR ( "HPE" ) OR ( medic* AND educat* ) OR ( medic* AND student ) OR ( medic* AND undergrad* ) OR ( medic* AND grad* ) OR ( medic* AND college ) OR ( medic* AND school ) OR ( pharma* AND educat* ) OR ( pharma* AND student* ) OR ( pharma* AND undergrad* ) OR ( pharma* AND grad* ) OR ( pharma* AND college ) OR ( pharma* AND school ) OR ( nurs* AND educat* ) OR ( nurs* AND student* ) OR ( nurs* AND undergrad* ) OR ( nurs* AND grad* ) OR ( nurs* AND college ) OR ( nurs* AND school ) OR ( dental AND educat* ) OR ( dental AND student* ) OR ( dental AND undergrad* ) OR ( dental AND grad* ) OR ( dental AND college ) OR ( dental AND school ) OR ( dentist* AND educat* ) OR ( dentist* AND student* ) OR ( dentist* AND undergrad* ) OR ( dentist* AND grad* ) OR ( dentist* AND college ) OR ( dentist* AND school ) OR ( "health science*" AND educat* ) OR ( "health science*" AND student* ) OR ( "health science*" AND undergrad* ) OR ( "health science*" AND grad* ) OR ( "health science*" AND college ) OR ( "health science*" AND school ) OR ( nutrition AND educat* ) OR ( nutrition AND student* ) OR ( nutrition AND undergrad* ) OR ( nutrition AND grad* ) OR ( nutrition AND college ) OR ( nutrition AND school ) OR ( physiotherap* AND educat* ) OR ( physiotherap* AND student* ) OR ( physiotherap* AND undergrad* ) OR ( physiotherap* AND grad* ) OR ( physiotherap* AND college ) OR ( physiotherap* AND school ) OR ( "physical therap*" AND educat* ) OR ( "physical therap*" AND student* ) OR ( "physical therap*" AND undergrad* ) OR ( "physical therap*" AND grad* ) OR ( "physical therap*" AND college ) OR ( "physical therap*" AND school ) OR ( "occupation* therap*" AND educat* ) OR ( "occupation* therap*" AND student* ) OR ( "occupation* therap*" AND undergrad* ) OR ( "occupation* therap*" AND grad* ) OR ( "occupation* therap*" AND college ) OR ( "occupation* therap*" AND school ) OR ( "public health" AND educat* ) OR ( "public health" AND student* ) OR ( "public health" AND undergrad* ) OR ( "public health" AND grad* ) OR ( "public health" AND college ) OR ( "public health" AND school ) OR ( midwife* AND educat* ) OR ( midwife* AND student* ) OR ( midwife* AND undergrad* ) OR ( midwife* AND grad* ) OR ( midwife* AND college ) OR ( midwife* AND school ) OR ( veterin* AND educat* ) OR ( veterin* AND student* ) OR ( veterin* AND undergrad* ) OR ( veterin* AND grad* ) OR ( veterin* AND college ) OR ( veterin* AND school ) OR ( paramedic* AND educat* ) OR ( paramedic* AND student* ) OR ( paramedic* AND undergrad* ) OR ( paramedic* AND grad* ) OR ( paramedic* AND college ) OR ( paramedic* AND school ) OR ( "speech and language" AND educat* ) OR ( "speech and language" AND student* ) OR ( "speech and language" AND undergrad* ) OR ( "speech and language" AND grad* ) OR ( "speech and language" AND college ) OR ( "speech and language" AND school ) OR ( "slt" AND educat* ) OR ( "slt" AND student* ) OR ( "slt" AND undergrad* ) OR ( "slt" AND grad* ) OR ( "slt" AND college ) OR ( "slt" AND school ) OR ( "physician assist*" AND educat* ) OR ( "physician assist*" AND student* ) OR ( "physician assist*" AND undergrad* ) OR ( "physician assist*" AND grad* ) OR ( "physician assist*" AND college ) OR ( "physician assist*" AND school ) OR ( "physician associate*" AND educat* ) OR ( "physician associate*" AND student* ) OR ( "physician associate*" AND undergrad* ) OR ( "physician associate*" AND grad* ) OR ( "physician associate*" AND college ) OR ( "physician associate*" AND school ) OR ( dietician AND educat* ) OR ( dietician AND student* ) OR ( dietician AND undergrad* ) OR ( dietician AND grad* ) OR ( dietician AND college ) OR ( dietician AND school ) OR ( dietetic AND educat* ) OR ( dietetic AND student* ) OR ( dietetic AND undergrad* ) OR ( dietetic AND grad* ) OR ( dietetic AND college ) OR ( dietetic AND school ) ) ) AND ( TITLE-ABS-KEY ( ( "generative artificial intelligen*" ) OR ( "gen ai" ) OR ( genai ) OR ( "generative ai" ) OR ( chatgpt ) OR ( "chat gpt" ) OR ( "large language model*" ) OR ( "llm" ) OR ( "Generative Pre Trained Transformer" ) OR ( "Generative PreTrained Transformer" ) OR ( gpt3 ) OR ( gpt4 ) OR ( gpt 3 ) OR ( gpt 4 ) ) ) AND PUBYEAR > 2017 AND PUBYEAR < 2026 |
| --- |

### Proquest

| noft(( ( biomedic* AND educat* ) OR ( biomedic* AND student* ) OR ( biomedic* AND undergrad* ) OR ( biomedic* AND grad* ) OR ( biomedic* AND college ) OR ( biomedic* AND school ) OR ( "health profession* educat*" ) OR ( "health care profession* educat*" ) OR ( "healthcare profession* educat*" ) OR ( "HPE" ) OR ( medic* AND educat* ) OR ( medic* AND student ) OR ( medic* AND undergrad* ) OR ( medic* AND grad* ) OR ( medic* AND college ) OR ( medic* AND school ) OR ( pharma* AND educat* ) OR ( pharma* AND student* ) OR ( pharma* AND undergrad* ) OR ( pharma* AND grad* ) OR ( pharma* AND college ) OR ( pharma* AND school ) OR ( nurs* AND educat* ) OR ( nurs* AND student* ) OR ( nurs* AND undergrad* ) OR ( nurs* AND grad* ) OR ( nurs* AND college ) OR ( nurs* AND school ) OR ( dental AND educat* ) OR ( dental AND student* ) OR ( dental AND undergrad* ) OR ( dental AND grad* ) OR ( dental AND college ) OR ( dental AND school ) OR ( dentist* AND educat* ) OR ( dentist* AND student* ) OR ( dentist* AND undergrad* ) OR ( dentist* AND grad* ) OR ( dentist* AND college ) OR ( dentist* AND school ) OR ( "health science*" AND educat* ) OR ( "health science*" AND student* ) OR ( "health science*" AND undergrad* ) OR ( "health science*" AND grad* ) OR ( "health science*" AND college ) OR ( "health science*" AND school ) OR ( nutrition AND educat* ) OR ( nutrition AND student* ) OR ( nutrition AND undergrad* ) OR ( nutrition AND grad* ) OR ( nutrition AND college ) OR ( nutrition AND school ) OR ( physiotherap* AND educat* ) OR ( physiotherap* AND student* ) OR ( physiotherap* AND undergrad* ) OR ( physiotherap* AND grad* ) OR ( physiotherap* AND college ) OR ( physiotherap* AND school ) OR ( "physical therap*" AND educat* ) OR ( "physical therap*" AND student* ) OR ( "physical therap*" AND undergrad* ) OR ( "physical therap*" AND grad* ) OR ( "physical therap*" AND college ) OR ( "physical therap*" AND school ) OR ( "occupation* therap*" AND educat* ) OR ( "occupation* therap*" AND student* ) OR ( "occupation* therap*" AND undergrad* ) OR ( "occupation* therap*" AND grad* ) OR ( "occupation* therap*" AND college ) OR ( "occupation* therap*" AND school ) OR ( "public health" AND educat* ) OR ( "public health" AND student* ) OR ( "public health" AND undergrad* ) OR ( "public health" AND grad* ) OR ( "public health" AND college ) OR ( "public health" AND school ) OR ( midwife* AND educat* ) OR ( midwife* AND student* ) OR ( midwife* AND undergrad* ) OR ( midwife* AND grad* ) OR ( midwife* AND college ) OR ( midwife* AND school ) OR ( veterin* AND educat* ) OR ( veterin* AND student* ) OR ( veterin* AND undergrad* ) OR ( veterin* AND grad* ) OR ( veterin* AND college ) OR ( veterin* AND school ) OR ( paramedic* AND educat* ) OR ( paramedic* AND student* ) OR ( paramedic* AND undergrad* ) OR ( paramedic* AND grad* ) OR ( paramedic* AND college ) OR ( paramedic* AND school ) OR ( "speech and language" AND educat* ) OR ( "speech and language" AND student* ) OR ( "speech and language" AND undergrad* ) OR ( "speech and language" AND grad* ) OR ( "speech and language" AND college ) OR ( "speech and language" AND school ) OR ( "slt" AND educat* ) OR ( "slt" AND student* ) OR ( "slt" AND undergrad* ) OR ( "slt" AND grad* ) OR ( "slt" AND college ) OR ( "slt" AND school ) OR ( "physician assist*" AND educat* ) OR ( "physician assist*" AND student* ) OR ( "physician assist*" AND undergrad* ) OR ( "physician assist*" AND grad* ) OR ( "physician assist*" AND college ) OR ( "physician assist*" AND school ) OR ( "physician associate*" AND educat* ) OR ( "physician associate*" AND student* ) OR ( "physician associate*" AND undergrad* ) OR ( "physician associate*" AND grad* ) OR ( "physician associate*" AND college ) OR ( "physician associate*" AND school ) OR ( dietician AND educat* ) OR ( dietician AND student* ) OR ( dietician AND undergrad* ) OR ( dietician AND grad* ) OR ( dietician AND college ) OR ( dietician AND school ) OR ( dietetic AND educat* ) OR ( dietetic AND student* ) OR ( dietetic AND undergrad* ) OR ( dietetic AND grad* ) OR ( dietetic AND college ) OR ( dietetic AND school ) ) ) AND noft(( ( "generative artificial intelligen*" ) OR ( "gen ai" ) OR ( genai ) OR ( "generative ai" ) OR ( chatgpt ) OR ( "chat gpt" ) OR ( "large language model*" ) OR ( "llm" ) OR ( "Generative Pre Trained Transformer" ) OR ( "Generative PreTrained Transformer" ) OR ( gpt3 ) OR ( gpt4 ) OR ( gpt 3 ) OR ( gpt 4 ) ) ) |
| --- |
